# Inflammation in Cystic Fibrosis

**DOI:** 10.1155/S096293519600021X

**Published:** 1996-04


Mediators of Inflammation 5, 121–143 (1996)

### Inflammation in Cystic Fibrosis

### International Symposium

**organized by**
**L'Association Française de Lutte contre la Mucoviscidose,**
**the German Mukoviszidose e.v.**
**and the Réseau Necker Mucoviscidose**
**under the patronage of**
**l'Institut National de la Santé et de la Recherche Médicale**
**at**
**La Maison de la Recherche, Paris, France**
**14–15 June 1996**

### Introduction

Chronic airway inflammation is a consistent finding in patients with cystic fibrosis (CF). Early bacterial colonization and infection of the airways with persisting pathogens such as *Staphylococcus aureus* and *Pseudomonas aeruginosa* are generally thought to provoke the activation of cells of the specific and nonspecific immune system and the migration of blood neutrophils to the lung. The consequences of chronic inflammation are decreasing lung function and premature death of CF patients. The mechanisms leading to inflammation in CF patients and the complex cellular interactions in inflammation mediated by a number of mediators including proteinases, reactive oxygen species and cytokines as well as their inhibitors are complex. Progress in understanding this important aspect of CF may be accelerated by discussions and collaborations between researchers with different skills. It is this aim which led to an international symposium on inflammation in CF where a number of relevant topics were discussed and the present knowledge summarized by experts in their fields. Such knowledge will hopefully result in more potent and selective anti-inflammatory therapeutic strategies, and thus increase life expectancy in CF.

Clinical and pathophysiological aspects of lung inflammation were discussed in detail. **Michel Aubier, Paris, France** focuses on compartimentalized lung inflammation in patients with pulmonary infections. Then, **Annick Clément, Paris, France** reports on recent studies showing that inflammation in CF may precede lung infection in contrast to the paradigm that infection precedes inflammation. If this finding can be further substantiated, the mechanisms are to be elucidated and new approaches to monitor pulmonary inflammation in very young infants are needed. Interestingly, the expression and/or regulation of inflammatory mediators from macrophages and epithelial cells may be different in normal individuals and CF patients. **Melvin Berger, Cleveland, USA** reports that CF bronchial epithelial cells contain much less IL-10 than those from normal healthy individuals, hypothesizing whether defective CFTR function could alter epithelial cell cytokine production. **Jean-Michel Dayer, Geneva, Switzerland** presents the complex network of pro-inflammatory cytokines and their inhibitors stressing on the concept of a possible dissociation between immunosuppressive and anti-inflammatory actions of cytokine inhibitors and its potential impact on therapeutic strategies. Damage and remodelling of CF airway epithelium caused by inflammation may also facilitate adhesion of *P. aeruginosa* as described by **Sophie Girod de Bentzmann, Reims, France**.

The mechanisms of neutrophil recruitment to the CF airway lumen, the release of lysosomal

© 1996 Rapid Science Publishers enzymes and the preventive strategies have been of major interest in this symposium. Based on experimental models of lung inflammation, **Peter Ward, Ann Arbor, USA** discusses the mediators for neutrophil recruitment and lung disease. **Jay Nadel, San Francisco, USA** describes a complex signalling process involving compounds from bacteria, the airway epithelium and the neutrophil causing chemotaxis and lysosomal enzyme release. **Elaine Tuomanen, New York, USA** reports on trials in which antibodies to CD18 or molecules which bind to receptors of CD18 blocked neutrophil migration. **Robert Stockley, Birmingham, UK** summarizes the current knowledge about the genes of the neutrophil serine proteinases, elastase, cathepsin G and proteinase-3 and discusses modulatory processes for elastase expression during cell differentiation. **Gerd Döring, Tübingen, Germany** reports on trials using aerosolized $\alpha_1$-proteinase inhibitor to suppress neutrophil elastase activity in CF airways.

Oxidative stress, as a consequence of oxidant/anti-oxidant imbalances, is another important topic of this symposium. **Véronique Witko-Sarsat, Paris, France** reviews the current knowledge on the mechanisms of neutrophil formation of reactive oxygen species and long-lived oxidants and presents recent data demonstrating that homozygote and heterozygote CF individuals overexpress neutrophil oxidative activity. **Frank J. Kelly, London, UK** demonstrates that biological markers of oxidative stress and antioxidant scavengers are present in plasma, urine and sputum of CF patients, suggesting that antioxidant therapy could reduce lung injury in CF. In contrast, **Dieter Worlitzsch, Tübingen, Germany** explains the absence of detectable hydrogen peroxide in the exhaled breath condensates of CF patients with the presence of high sputum concentrations of myeloperoxidase and catalase.

Based on the clinical experience, conventional anti-inflammatory drugs, presented by **Michael Konstan, Cleveland, USA** may only reduce the inflammatory manifestations of the airways; therefore gene therapy is discussed as an alternative approach to prevent lung inflammation. **Catherine Figarella, Marseille, France** reports on the expression and function of CFTR in CF and normal airways. **Patricia Lemarchand, Paris, France** discusses the hurdles of gene replacement therapy for defective CFTR and for oxidative stress. *In vitro* studies by **Aleksander Edelman, Paris, France** reveal that cytokines and aspirin may downregulate CFTR gene expression suggesting that inflammation may impair gene therapy. **Gabriel Bellon, Lyon, France** reports on the results of a CFTR gene replacement trial in six CF patients and **Claire Danel, Paris, France** focuses on the problem of lung inflammation consecutive to gene transfer.

In conclusion, Inflammation in Cystic Fibrosis closely reflects the hostile face of Janus chosen by the Editor-in-Chief, Ivan Bonta, as 'the symbol *par excellence*' for *Mediators of Inflammation*. We hope that the basic and novel aspects on the role of inflammation in CF and therapeutic strategies discussed in this symposium will have an important impact on the management of chronic inflammatory lung disease in CF patients.

**Béatrice Descamps-Latscha, MD, PhD**
*INSERM U25, Hôpital Necker, Paris, France*

**Gerd Döring, PhD**

*Hygiene-Institut, Tübingen, Germany*

**Pierre Galanaud, MD**

*INSERM U131, Hôpital Antoine Béclère, Clamart, France*

**Gérard Lenoir, MD**

*Hôpital Necker, Paris, France*

**Jean Navarro, MD**

*Hôpital Robert Debré, Paris, France*

**Laurence Schaffar, MD, PhD**
*INSERM, Paris, France*

ACKNOWLEDGEMENTS. We are most grateful to Professor Ivan L. Bonta, Editor-in-Chief of *Mediators of Inflammation*, who has accepted to publish the abstracts of the Symposium in a bilingual version and to Rapid Science Publishers, who generously offered this regular issue of the journal and accelerated its publication in order to make it available for the Symposium.

### List of Participants

Michel Aubier, MD, Paris, France; Gabriel Bellon, MD, Lyon, France; Melvin Berger, MD, Cleveland, USA; Annick Clément, MD, Paris, France; Claire Danel, MD, Paris, France; Jean-Michel Dayer, MD, Geneva, Switzerland; Béatrice Descamps-Latscha, MD, Paris, France; Gerd Döring, PhD, Tübingen, Germany; Daniel Dusser, MD, Paris, France; Aleksander Edelman, PhD, Paris, France; Catherine Figarella, MD, Marseille, France; Pierre Galanaud, MD, Clamart, France; Eva Giesen, PhD, Paris, France; Sophie Girod de Bentzmann, PhD, Reims, France; Michel Goossens, MD, Créteil, France; Frank J. Kelly, MD, London, UK; Michael Konstan, MD, Cleveland, USA; Patricia Lemarchand, MD, Paris, France; Gérard Lenoir, MD, Paris, France; Jay Nadel, MD, San Francisco, USA; Jean Navarro, MD, Paris, France; Laurence Schaffar, MD, Paris, France; Robert A. Stockley, MD, Birmingham, UK; Elaine Tuomanen, MD, New York, USA; Peter Ward, MD, Ann Arbor, USA; Véronique Witko-Sarsat, PhD, Paris, France; Dieter Worlitzsch, MD, Tübingen, Germany.

### Abstracts

### Compartmentalized lung inflammation in response to infection

*Michel Aubier*
Service de Pneumologie, Unité INSERM 408, Hôpital Bichat, 46 rue Henri Huchard, 75018 Paris, France

Normally, bacteria are prevented from reaching the alveoli by several defense mechanisms located along the normal upper airways. Furthermore, bacteria reaching the alveoli are usually phagocytized and killed by resident macrophages.[1] When normal clearance mechanisms are overwhelming, a complex response develops. At a peripheral tissue site, invasion by a bacterial pathogen produces an inflammatory response that is accompanied by the activation of local macrophages by bacterial products such as endotoxin and cell-wall components. The early stage of infection may be characterized by the generation of multiple signals, including a cascade of endogenous host mediators triggered by bacterial products. Among these mediators, cytokines and particularly tumour necrosis factor-α (TNF-α), interleukin-1β (IL-1β), interleukin-6 (IL-6) and IL-8 are thought to mediate many host responses to bacterial infection. These cytokines are involved in the response to infection, including the activation of immune cells leading to the production of specific antibodies. They are also involved in the recruitment and activation of monocytes and neutrophils to areas of infection following activation of endothelial cells.

Although excessive cytokine production during severe infection has many deleterious effects that may lead to death,[2] various animal models support the beneficial role of cytokines in natural host resistance to local infection. This suggests that locally produced cytokines may contribute to eradicating the invading pathogen.[3] Indeed, some cytokines appear to have more local effects within their tissue of origin than systemic effects.[4–6]

In this regard, several human and animal studies have demonstrated significant differences between the inflammatory response in intravascular models of infection and in localized bacterial infection such as meningitis.[4,5] However, little is known about the *in situ* inflammatory response developing in the human lung during localized bacterial infection.

In two recent studies,[7,8] the *in situ* inflammatory response developing in the human lung during a localized bacterial infection was studied in 15 patients with unilateral community-acquired pneumonia (CAP). The local response in the involved lung was compared with that in the controlateral, non-involved lung as well as with the systemic blood response. Eight healthy volunteers served as control subjects. Concentrations of tumour necrosis factor-α (TNF-α), interleukin-1β (IL-1β), and interleukin-6 (IL6) and IL-8 were measured by ELISA in bronchoalveolar lavage (BAL) fluids ($n = 15$), serum ($n = 15$), and alveolar macrophage and monocyte culture supernatant ($n = 8$). The concentrations of TNF-α, IL-β, IL6 and IL-8 in BAL fluid were significantly higher in the involved lung than in the paired non-involved lung ($p \leqslant 0.01$) or in healthy subjects ($p \leqslant 0.02$, $p \leqslant 0.01$, $p \leqslant 0.001$, $p \leqslant 0.0001$, respectively). Serum IL-6 concentrations were higher in patients than in control subjects, whereas IL-1β, TNF-α and IL-8 concentrations did not differ in the two groups. Alveolar macrophages from the involved lung spontaneously released higher concentrations of IL-1β, IL-6, and TNF-α ($p \leqslant 0.05$) than did macrophages from the non-involved lung, which served as control. However, macrophages were hyporesponsive in terms of cytokine production to further stimulation by lipopolysaccharide (LPS) in the noninvolved and involved lung compared with controls, whereas peripheral blood monocytes were not.

These data in humans provide strong evidence that during unilateral CAP, the inflammatory response is compartmentalized within the human lung and is limited to the site of infection. Although TNF-α and IL-1β are believed to have synergistic effects that induce lung damage and leakage through the capillary wall,[9] a beneficial role has also been ascribed to cytokines in limiting bacterial spread.[10] The localized and compartmentalized production of cytokines within the lung in unilateral pneumonia confirms that these substances play an important role in the local lung response to infection. Concomitantly, the observed hyporeactivity in LPS-induced cytokine synthesis might be the result of a homeostatic process that would limit the local inflammatory response and thus protect the lung from the deleterious effects of excessive cytokine production, which might otherwise contribute to tissue damage and particularly of alveolar epithelial cells.

Among the latter, type II alveolar epithelial cells (ATII cells) have been shown to play an active role in the regulation of the inflammatory reaction within alveolar space. Indeed, ATII cells are ideally located to have a role in modulating immunologic activity in the alveolar space and both *in vitro* and *in vivo* data suggest that ATII cells could participate in the intra-alveolar cytokine network by secreting interleukin-8 (IL-8),[11] interferon,[12] monocyte chemoattractant protein-1,[13] transforming growth factor beta[14] platelet-derived growth factor[15,16] and granulocyte monocyte colony-stimulating factor[17] and more recently IL-6 under appropriate stimulation.[18]

The latter cytokine has been shown to exert some anti-inflammatory functions. *In vitro*, at pathologic concentrations, Il-6 inhibits T cell responses via the activation of macrophages that secrete TGF-β, a cytokine that impairs both T cell and thymocyte proliferative responses.[18] Moreover, IL-6 downregulates IL-1β and TNFα gene expression in human activated monocytes,[19] modulates the synthesis of α1-anti-trypsin *in vitro* in human mononuclear phagocytes[20] and induces a dysfunction of natural killer functions of lymphocytes.[21] *In vivo*, in rats, intra-tracheal IL-6 partially inhibited the neutrophil alveolitis induced by intra-tracheal LPS.[22]

Thus, it appears that cellular communication between immune and non-immune cells is an essential process in the initiation maintenance, and resolution of the intra-alveolar inflammatory response. A better understanding of the processes which regulate the inflammatory processes within the alveolar space may lead to a more rational and new therapeutic approach in patients with infectious or non-infectious alveolitis.

### References

1. Sibille Y, Reynolds HY. Macrophages and polymorphonuclear neutrophils in lung defense and injury. *Am Rev Respir Dis* 1990; **141**: 471–501.
2. Waage A, Brandtzaeg P, Halstensen A, Kierulf P, Espevik T. The complex pattern of cytokines in serum from patients with meningococcal septic shock. *J Exp Med* 1989; **169**: 333–338.
3. Havell EA. Evidence that tumour necrosis factor has an important role in anti-bacterial resistance. *J Immunol* 1989; **143**: 2894–2899.
4. Rugo HS, O'Hanley P, Bishop AG *et al.* Local cytokine production in a murine model of *Escherichia coli* pyelonephritis. *J Clin Invest* 1992; **89**: 1032–1039.
5. Waage A, Halstensen A, Shalaby R, Brandtzaeg P, Kierulf P, Espevik T. Local production of tumor necrosis factor-α, interleukin-1, and interleukin-6 in meningococcal meningitis. *J Exp Med* 1989; **170**: 1859–1867.
6. Wimott RW, Kassab JT, Kilian PL, Benjamin WR, Douglas SD, Wood RD. Increased levels of interleukin-1 bronchoalveolar washings from children with bacterial pulmonary infections. *Am Rev Respir Dis* 1990; **142**: 365–368.
7. Dehoux MS, Boutten A, Ostinelli J, *et al.* Compartmentalized cytokine production within the human lung in unilateral Pneumonia. *Am J Respir Crit Care Med* 1994; **150**: 710–716.
8. Boutten A, Dehoux MS, Seta N, *et al.* Compartmentalized IL-8 and elastase release within the human lung unilateral pneumonia. *Am J Respir Crit Care Med* 1996; **153**; 336–342.
9. Okusawa S, Gelfand JA, Ikejima T, Connoly RJ, Dinarello CA. Interleukin-1 induces a shock-like state in rabbits: synergism with tumor necrosis factor and the effect of cyclooxygenase inhibition. *J Clin Invest* 1988; **81**: 1162–1172.
10. Munoz C, Carlet J, Fitting C, Misset B, Bleriot JP, Cavaillon J. Dysregulation of *in vitro* cytokine production by monocytes during sepsis. *J Clin Invest* 1991; **88**: 1747–1754.
11. Standiford TJ, Kundel SL, Basha MA, *et al.* Interleukin-8 gene expression by a pulmonary epithelial cell line. A model for cytokine networks in the lung. *J Clin Invest* 1990; **86**: 1945–1953.
12. Hahon N, Castranova V. Interferon production in rat type II pneumocytes and alveolar macrophages. *Exp Lung Res* 1989; **15**: 429–445.
13. Standiford TJ, Kundel SL, Phan SH, Rollins BJ, Strieter RM. Alveolar macrophage-derived cytokines induce monocyte chemoattractant protein-1 expression from human pulmonary type II-like epithelial cells. *J Biol Chem* 1991; **266**; 9912–9918.
14. Khalil N, O'Connor RN, Unruch HW, *et al.* Increased production and immuno-histochemical localization of transforming growth factor beta in idiopathic pulmonary fibrosis. *Am J Respir Cell Mol Biol.* 1991; **5**: 155–162.
15. Antoniades HN, Bravo MA, Avila T, Galanopoulos T, Neville-Golden J. Platelet-derived growth factor in idiopathic pulmonary fibrosis. *J Clin Invest* 1990; **86**: 1055–1064.
16. Vignaud JM, Allam M, Martinet N, Pech M, Plenat F, Martinet Y. Presence of platelet-derived growth factor in normal and fibrotic lung is specifically associated with interstitial macrophages, while both interstitial macrophages and alveolar epithelial cells express the C-sis proto-oncogene. *Am J Respir Cell Mol Biol* 1991; **5**: 531–538.
17. Tazi A, Bouchonnet F, Grandsaigne M, Boumsell L, Hance AJ, Soler P. Evidence that granulocyte-macrophage colony stimulating factor regulates the distribution and differentiated state of dendritic cells/Langherhans cells in human lung and lung cancers. *J Clin Invest* 1991; **91**: 566–576.
18. Crestani B, Cornillet P, Dehoux M, Rolland C, Guenounou M, Aubier M. Alveolar type II epihelial cells produce interleukin-6 *in vitro* and *in vivo*. Regulation by alveolar macrophage secretory products. *J Clin Invest* 1994; **94**: 731–740.
19. Zhou D, Munster A, Winchurch RA. Pathologic concentrations of interleukin 6 inhibit T cell responses via induction of activation of TGF-β. *FASEB* 1991; **5**: 2582–2585.
20. Schindler R, Mancilla J, Endres S, Ghorbani R, Clark SC, Dinarello CA. Correlations and interactions in the production of interleukin 6 (IL-6), IL-1, and tumor necrosis factor (TNF) in human blood mononuclear cells: IL-6 suppresses IL-1 and TNF. *Blood* 1990; **75**: 40–47.
21. Perlmutter DH, May LT, Sehgal PB. Interferon β2/interleukin-6 modulates synthesis of α1-antitrypsin in human mononuclear phagocytes and in human hepatoma cells. *J Clin Invest* 1989; **84**: 138–144.
22. Tanner J, Tosato G. Impairment of natural killer functions by interleukin-6 increases lymphoblastoid cell tumorigenicity in athymic mice. *J Clin Invest* 1991; **88**: 239–247.
23. Ulich TR, Yin S, Guo K, Yi ES, Remick D, Del Castillo J. Intratracheal injection of endotoxin and cytokines. II. Interleukin-6 and transforming growth factor beta inhibit acute inflammation. *Am J Pathol* 1991; **138**: 1097–1101.

### Inflammatory processes in cystic fibrosis

*Annick Clément*

Département de Pneumologie Pédiatrique, Hôpital Armand Trousseau, Paris, France

The pulmonary manifestations of cystic fibrosis (CF) result from chronic bronchopulmonary infection leading to respiratory insufficiency by obstructive lung disease. Colonization of the lower respiratory tract by bacteria such as *Pseudomonas aeruginosa* has been thought initially to be a critical event in the initiation and development of inflammatory processes. However, it is likely that the cascade associated with disease progression involves first pulmonary inflammation and subsequently infection of the lower respiratory tract which may therefore result from the inflammatory insult.

The current view of the pathogenesis of lung disease in CF with initial inflammation is supported by recent studies. Controlled analyses of bronchoalveolar lavage fluid from infants with CF have documented the presence of increased inflammatory parameters including neutrophils counts, activity of free neutrophil elastase and IL-8 levels. Interestingly, these markers of pulmonary inflammation were found even in the absence of common bacterial CF-related pathogens, of respiratory viruses and of fungi.

These recent observations raise two issues. First, understanding of the mechanisms associated with initiation of the inflammatory response in infants with CF appears to be critical for the development of new therapeutic strategies. Several hypotheses can be discussed. The cells most involved in the onset of inflammatory response are the local macrophages. From recent studies, it is believed that airway and alveolar macrophages are an important source of IL-8 in CF. The reason for an increased expression of IL-8 by macrophages is still unclear. It may be induced by external factors such as the abnormal mucus which accumulates in the lower airways in CF. It may also reflect an altered function of the macrophages. Indeed, expression of the cystic fibrosis transmembrane conductance regulator gene has been documented in macrophages and this gene seems to play a role in the control of several cellular functions including cytokine production and secretion. Another element that may be involved in the amplification of the inflammatory response is the respiratory epithelium. The cells that compose the epithelium of the airways and of the alveoli are known to have the ability of producing a wide range of inflammatory mediators. They serve as part of the local immune system, providing structures and functions crucial for the maintenance of normal pulmonary function. It is now well established that alteration of the pulmonary epithelium plays an important role in the pathogenesis of many respiratory diseases. In CF, epithelial cells can participate in the progressive destruction of airways and lung tissue through interactions with bacteria and their products, as well as through interactions with cells present in the airways. These cell–cell interactions are certainly involved in the up-regulation of inflammatory mediators released by the respiratory cells. However, the mechanisms leading to altered expression of the molecules of the inflammatory response as well as the role of cystic fibrosis transmembrane conductance regulator gene in this control remain poorly defined.

The second issue relates to the need of early markers of inflammatory response in infants. Indeed, if inflammation is an initial event in disease progression, efforts should be made to define tools of interest for evaluation of local inflammation in very young children. In this view, bronchoalveolar lavage is certainly of great interest; however, its relative invasiveness precludes its repeated utilization during the follow-up of the patients. Clearly, new approaches of pulmonary inflammation evaluation in infants are now required in order to optimize the therapeutic strategies which could be proposed. These approaches should include studies of sputum samples or of aspirates from the upper airways.

### Experimental models of lung inflammation

*Peter A. Ward*
Department of Pathology, The University of Michigan Medical School, Ann Arbor, Michigan, USA

Animal models of inflammation have provided important information regarding mechanisms by which inflammatory cellular responses occur and the manner by which tissue injury develops. The most comprehensive understanding of acute lung inflammatory injury in rats has come from the use of the IgG immune complex model in which alveolar deposition of immune complexes leads to *in situ* complement activation. These complexes, together with complement activation products, activate lung macrophages to generate the early response cytokine, TNF-α and IL-1. Concurrently, these cells generate the C-C chemokine MIP-1α, which seems to have autocrine positive feed-back effects, enhancing macrophage production of TNF-α and IL-1. The primary role of these two cytokines is to cause upregulation of lung vascular ICAM-1 and E-selectin. Through a sequence of adhesion promoting-interactions, these adhesion molecules facilitate the influx of neutrophils into the alveolar compartment.

Neutrophil recruitment requires E-selectin, ICAM-1 and CD11a/CD18 within the vascular compartment and CD11b/CD18 and ICAM-1 within the airway compartment. The chemotactic influx of neutrophils into lung is mediated at least in part, by a C-X-C chemokine, MIP-2. Injury to the alveolar and vascular walls occurs via production of nitric oxide (NO) generated by inducible NO synthase (iNOS), which has two sources in lung: alveolar Type II epithelial cells and macrophages. Induction of iNOS appears to be regulated by three cytokines: TNF-α IL-1 and interferon-gamma. Lung matrix damage occurs following release from macrophages and neutrophils of serine proteinases and metalloproteinases.

These lung inflammatory reactions are self-limited, apparently because of concomitant appearance of IL-10 which suppresses macrophage production of TNF-α and IL-10. Endogenous production of IL-10 has been shown by the appearance of mRNA and IL-10 protein in lung following deposition of immune complexes. Blocking of endogenous IL-10 leads to a significant increase in lung injury. These data demonstrate that lung inflammatory reactions are mediated by a variety of pro-inflammatory cytokines and regulated by at least one anti-inflammatory cytokine (IL-10).

### Process of lesion and repair of airway epithelium

*Sophie Girod de Bentzmann*
INSERM U 314, CHR Maison Blanche, Reims, France

In the normal human respiratory tract, the pseudostratified airway epithelium is protected from aerocontaminants including viruses, bacteria and pollutants, by the combination of a mucus gel layer and of a mucociliary clearance mechanism by means of the ciliary activity.

In pathological conditions, such as in cystic fibrosis (CF), the mucociliary clearance is impaired due, in particular, to hypersecretion and hyperviscosity of respiratory mucus. The defective mucociliary clearance in CF, associated with marked chronic inflammation creates local propitious conditions for epithelial injury. Epithelial injury can vary from epithelial remodelling, including loss of ciliated cells to epithelial shedding with focal basal cells still attached to the basement membrane.

Various molecules, bacteria or viruses have been extensively described as being able to induce such epithelial damages. These include ciliostastic effects,[1] mucous hyperplasia,[2] and epithelial shedding.[3,4]

Apart from bacterial or viral products, local immune response to bacterial or viral infections can lead to increased amounts of serum proteases in the extracellular fluid able to induce epithelial remodelling. It has been clearly shown that human leucocyte elastase can induce a wide variety of epithelial remodelling including mucous to serous cell conversion,[5] mucous cell hyperplasia[6] and epithelial shedding.[7] In CF, chronic airway infection associated with chronic airway inflammation represent sources of epithelial injury. Baltimore *et al.*[8] have described in an extensive histopathologic study, the intense remodelling of airway epithelium, in CF patients. Lesion of airway epithelium, which represents the ultimate degree of epithelial damage is followed by repair. The repair process of airway epithelium has been described by Zahm *et al.*[9] in an *in vitro* model of wound repair of airway epithelium. Depending on the size of the wound, airway epithelial cells are able to spread, migrate and proliferate[10] in order to restore the integrity of the epithelial barrier.[11] Denuded basement membrane and in particular laminin[12] represent sites of adherence for *Pseudomonas aeruginosa*, one of the most important pathogens recovered in CF. Airway epithelial cells engaged in the repair process represent also important sites of adherence for *P. aeruginosa*. We have recently described the significantly increased adherence of *P. aeruginosa* to airway epithelial cells which spread at the edge of the wound from CF and non-CF origins. Phenotypic alterations of repairing airway epithelial cells, spreading at the edge of the wound, are responsible for the overexpression of epithelial receptors for *P. aeruginosa* adherence. Among epithelial receptors, asialylated membrane glycolipids such as asialo GM1 were recovered at the surface of repairing airway epithelial cells acting as receptors for *P. aeruginosa* adherence. Although asialo GM1 represents a site of adherence at the surface of CF repairing airway epithelial cells, we identified asialo GM1 at the surface of non-CF repairing airway epithelial cells, acting as receptors for *P. aeruginosa*.[13] Moreover, the apical asialo GM1 expression on repairing airway epithelial cells decreases along the repair process. This suggests that the primary defect of CFTR in CF cannot represent the unique explanation for *P. aeruginosa* adherence to airway epithelial cells. Moreover, airway epithelial cells actively synthesize and secrete fibronectin during repair[14] which also represents a site of adherence for *P. aeruginosa*.[15] We are currently identifying the FN receptor on repairing airway epithelial cells. We hypothesize that dramatic phenotypic alterations of airway epithelial cells during lesion and repair widely contribute to chronic airway infection with *P. aeruginosa* in CF. Moreover, the susceptibility of repairing airway epithelium to *P. aeruginosa* adherence decreases along the repair process[16] in parallel to asialo GM1 expression, suggesting that immediately after wounding, the onset of repair of the airway epithelium represents a critical phase during which it would be useful to protect the regenerating airway epithelium.

### References

1. Kanthakumar K, Taylor G, Tsang KWT, *et al.* Mechanisms of action of *Pseudomonas aeruginosa* pyocyanin on human ciliary beat *in vitro. Infect Immun* 1993; **61**: 2848–2853.
2. Huang HT, Haskell A, MacDonald DM. Changes in epithelia secretory cells and potentiation of neurogenic inflammation in the trachea of rats with respiratory tract infections. *Anat Embryol* 1989; **180**: 325–341.
3. Bajolet-Laudinat O, Girod-de Bentzmann S, Tournier JM, *et al.* Cytotoxicity of *Pseudomonas aeruginosa* internal lectin PA I on respiratory epithelial cells in primary culture. *Infect Immun* 1994; **62**: 4481–4487.
4. Plotkowski MC, Puchelle E, Beck G, Jacquot J, Hannoun C. Adherence of type I *Streptococcus pneumonia* to tracheal epithelium of mice infected with influenza A/PRS virus. *Am Rev Respir Dis* 1986; **134**: 1040–1044.
5. Marchand V, Tournier JM, Polette M, Hannion X, Puchelle E. Effect of leucocyte elastase on the expression of antileucoprotease by human adult nasal surface epithelial cells in primary culture. *Eur Resp J* 1995; **8**: suppl. 19: 1365.
6. Snider GL, Lucey EC, Christensen PJ, Stone J, *et al.* Emphysema and bronchial secretory cell metaplasia induced in hamsters by human neutrophil products. *Am Rev Respir Dis* 1984; **129**: 155–160.
7. Plotkowski MC, Beck G, Tournier JM, Bernardo-Filtro M, Marques EA, Puchelle E. Adherence of *Pseudomonas aeruginosa* to respiratory epithelium and the effect of leucocyte elastase. *J Med Microbiol* 1989; **30**: 285–293.
8. Baltimore R, Christie C, Walker Smith G. Immunohistopathologic localization of *Pseudomonas aeruginosa* in lung from patients with cystic fibrosis. *Am Rev Respir Dis* 1989; **140**: 1650–1661.
9. Zahm JM, Chevillard M, Puchelle E. Wound repair of human surface respiratory epithelium. *Am J Respir Cell Mol Biol* 1991; **5**: 242–248.
10. Zahm JM, Kaplan H, Doriot F, *et al.* Cell migration and proliferation during the *in vitro* wound repair of the respiratory epithelium. *Micros Res Tech* 1996, in press.
11. Hérard AL, Zahm JM, Pierrot D, Hinnrasky J, Fuchey C, Puchelle E. Epithelial barrier integrity during *in vitro* wound repair of the airway epithelium. Submitted to *Am J Respir Cell Mol Biol* 1996, in press.
12. Plotkowski MC, Tournier JM, Puchelle E. *Pseudomonas aeruginosa* strains possess specific adhesins for laminin. *Infect Immun* 1996; **64**: 600–605.
13. de Bentzmann S, Roger P, Dupuit F, *et al.* Asialo GM1 as a receptor for *Pseudomonas aeruginosa* adherence to regenerating respiratory epithelial cells. *Infect Immun* 1996, in press.
14. Zahm JM, Hérard AL, Pierrot D, Hinnrasky J, Sheppard D, Puchelle E. Role of fibronectin and its a5b1 integrin receptor during respiratory epithelium wound repair. *Am J Resp Crit Care Med* 1995; **151(4)**: A561.
15. Plotkowski MC, Filho MB, Meirelles MN, Tournier JM, Puchelle E. *Pseudomonas aeruginosa* binds to soluble cellular fibronectin. *Curr Microbiol* 1993; **26**: 91–95.
16. de Bentzmann S, D'Allessandro F, Zahm JM, Pierrot D, Plotkowski MC, Puchelle E. Kinetics of *Pseudomonas aeruginosa* adherence during respiratory epithelial wound repair. *Eur Respir J* 1995; **8(19)**: 42S.

### CFTR expression in the airways: which cell is the target of the disease?

*Catherine Figarella and Marc Merten*
Groupe de Recherche sur les Glandes Exocrines, Faculté de Médecine, 27 Boulevard Jean Moulin, 13385 Marseille, Cedex 5, France

Cystic fibrosis (CF) lung disease is characterized by inadequate clearance of airway secretion and increased mucus production causing obstructive lung disease and chronic bronchial infections leading to progressive respiratory failure and death. The running hypothesis, by which mutations in the cystic fibrosis transmembrane conductance regulator (CFTR) gene lead to a defect in mucociliary clearance, is one where the defects in ion transport (decrease of chloride conductance) found in epithelia of conducting airways lead to dehydration of mucus, insipissation of secretions and defective clearance of inhaled pathogens. This in turn might explain why CF airways are liable to infection but does not explain the persistant colonization with *Pseudomonas aeruginosa* and the excessive mucous production. For instance it is known that patients with 'immotile cilia syndrome' have a more profound slowing of airway mucus transport than that seen in CF, but they suffer only from mild airway infections.

Critical to an understanding of the basic molecular pathology of the CF airway, is a definition of the normal expression of CFTR in the human lung. In a pioneer study, Engelhardt *et al.*, using *in situ* hybridization and immunochemistry, found a very low expression of CFTR in superficial epithelia of the proximal airways but a predominant site of CFTR expression in submucosal glands.[1] In these glands, high levels of CFTR-RNA and protein are found in all cells of serous tubules of the distal gland, and in 1–3% of cells in the more proximal collecting ducts. Using immunogold electron microscopy, Jacquot *et al.* demonstrated further that CFTR protein was more specifically associated with the secretory granules of glandular serous cells.[2] Later, CFTR was also detected in a subpopulation of epithelial cells at every level of the distal lung, including proximal, terminal, and respiratory bronchioles and the alveoli but with a substantial variation in the level of CFTR expression between samples.[3]

The fact that the highest level of CFTR expression was found in submucosal glands, is not surprising if we consider that the major problem in CF is an alteration of the rheology of mucus, and that these glands have long been recognized as being the main secretory structures in the mucosa of the bronchotracheal tree. In humans, the glandular volume is estimated to be about 4 ml, the volume of mucus cells present in the airways surface epithelium being 1/40 of this value. The role of submucosal glands in the CF pulmonary disease does not seem to be in doubt and the fact that the lungs of knockout mice, which lack CFTR entirely and normally have very few submucosal glands, are minimally affected[4] supports this hypothesis.

We recently characterized a primary cell culture model of human tracheal gland serous cells (HTGS), which, studied at the third passage, respond to cholinergic and adrenergic agonists and synthesize serous secretory markers such as the secretory leukocyte proteinase inhibitor as well as high molecular-weight glycoproteins.[5,6] These cells do express CFTR mRNA and contain low conductance CFTR-like chloride selective channels.[7] We could demonstrate that cultured CF-HTGS cells are subjected to an important defect in the regulation of the secretory process, since we observed in these cells a constitutive hypersecretion of proteins and a hyporesponsiveness to the two physiological neurotransmitters: acetylcholine and norepinephrine.[8] These defects are specific of CF-HTGS cells and were not found in cultured HTG cells derived from patients with other inflammatory diseases like sarcoidose or chronic obstructive pneumopathological disease (COPD). This observation indicates that in cystic fibrosis, besides ionic defects, there is a defect in protein secretion leading us to suspect a more general defect in the secretory process. In addition, our preliminary results show that normal HTGS cells cultured in the presence of the LPS of *P. aeruginosa*, behave in the same way as CF-HTGS cells, strongly suggesting that CF cells could be constitutively in an inflammatory state.

To conclude, many arguments, be they genetic, molecular or electrophysiologic, are now in favour of the theory that glandular tracheal cells are a privileged target of cystic fibrosis. The fact that their cellular mechanisms are not completely understood and that these cells are profoundly lodged in the mucosa will most likely influence the approaches of somatic gene therapy for the CF lung.

### References

1. Engelhardt JF, Yankaskas JR, Ernst SA, *et al.* Submucosal glands are the predominant site of CFTR expression in the human bronchus. *Nature Genet* 1992; **2**: 240–247.
2. Jacquot J, Puchelle E, Hinnrasky J, *et al.* Localization of the cystic fibrosis transmembrane conductance regulator in airway secretory glands. *Eur Respir J* 1993; **6**: 169–176.
3. Engelhardt JF, Zepeda M, Cohn JA, Yankaskas JR, Wilson JM. Expression of the cystic fibrosis gene in adult human lung. *J Clin Invest* 1994; **93**: 737–749.
4. Snouwaert JN, Brigman KK, Latour AM, *et al.* An animal model for cystic fibrosis made by gene targeting. *Science* 1992; **257**: 1083–1088.
5. Tournier JM, Merten MD, Meckler Y, Hinnrasky J, Fuchey C, Puchelle E. Culture and characterization of human tracheal gland cells. *Am Rev Respir Dis* 1990; **141**: 1280–1288.
6. Merten MD, Tournier J-M, Meckler Y, Figarella C. Secretory proteins and glycoconjugates synthesized by human tracheal gland cells in culture. *Am J Respir Cell Mol Biol* 1992; **7**: 598–605.
7. Becq F, Merten MD, Voelckel MA, Gola M, Figarella C. Characterization of cAMP dependent CFTR-chloride channels in human tracheal gland cells. *FEBS Letters* 1993; **321**: 73–78.
8. Merten MD, Figarella C. Constitutive hypersecretion and insensitivity to neurotransmitters by cystic fibrosis tracheal gland cells. *Am J Physiol* 1993; **264**: L93–L99.

### Mechanisms of neutrophil recruitment and activation during lung infection with *Pseudomonas aeruginosa*

*Jay A. Nadel*
University of California, San Francisco Cardiovascular Research Institute, San Francisco, CA, USA

The airway submucosal glands and surface goblet cells normally provide protective mechanisms against inhaled irritants. In chronic diseases of the airways, mucus hypersecretion occurs and is an important cause of airway obstruction in cystic fibrosis (CF). Large numbers of neutrophils and high concentrations of catalytically active neutrophil proteases exist in CF sputum, and neutrophil elastase is the most potent secretagogue ever examined. Thus, sputum from patients with CF is capable of stimulating secretion from gland cells in culture even when the sputum is diluted 30 000-fold! These findings implicate neutrophil elastase as a potentially important cause of airway hypersecretion. If neutrophil elastase is a cause of the hypersecretion, how is the elastase released from neutrophils? In the case of the goblet cells in the lumen, dead neutrophils are, at least in part, the probable cause of secretion, because CF sputum contains high concentrations of active neutrophil elastase.

In the case of gland cells, elastase in the lumen does not cause secretion and intact neutrophils do not normally (or easily) release significant elastase into the medium. I suggest that a complex signalling process involving the surface of the secretory cells activates second messengers in the neutrophil, causing exocytosis of elastase-containing granules in close contact with the secretory cell surface. What is the mechanism of neutrophil recruitment in CF airways? CF sputum contains high concentrations of interleukin-8 (IL-8) and a blocking antibody to IL-8 inhibits substantially the chemotactic activity of CF sputum, suggesting that IL-8 plays a significant role in neutrophil recruitment in CF.

In CF, *Pseudomonas aeruginosa* (PA) is an important infecting organism, and the supernatant of PA organisms causes time-dependent production of IL-8 in airway epithelium. Incubation of PA supernatant with normal airways obtained from lung transplant donors resulted in IL-8 expression selectively in the airway epithelium. To examine whether feedback mechanisms perpetuate or exaggerate IL-8 expression and neutrophil recruitment, we performed animal studies *in vivo*. We cloned and expressed IL-8 in dogs and studied IL-8 expression in a bypassed segment of trachea. PA supernatant caused early selective expression of IL-8 in airway epithelium, and this was due to a novel, small molecular weight, water soluble product from PA. Several hours later, IL-8 was strongly expressed in the recruited neutrophils, and this was due to PA lipopolysaccharide (LPS). Thus, neutrophil recruitment is potentiated. In natural infection, this would continue until the neutrophils killed all PA bacteria, and then the mechanism would be rapidly turned off.

In summary, neutrophil recruitment and release of products is a complicated process. Epithelial and neutrophil products form a positive feed-back system (which is an advantage to the normal host but apparently disadvantageous to CF patients). This inflammatory cascade provides multiple opportunities for potential therapeutic intervention.

### Prevention of leukocyte diapedesis in bacterial pneumonia

*Elaine Tuomanen*
Rockefeller University, New York, USA

While it is well accepted that endotoxin is the major noxious component of gram negative bacteria, it is less well appreciated that cell walls are equally inflammatory in the context of gram positive disease. The signs and symptoms of pneumonia induced by cell walls mimic that of living bacteria in animal models.[1] Clinical strains and their isogenic lab derivatives which have defects in release of cell wall fragments induce an attenuated pattern of disease.[2] These cell wall fragments are potently bioactive with some being specifically toxic to pulmonary epithelial cells,[3] while others induce the influx of leukocytes or increased permeability of the pulmonary endothelium.[4] Given that antibiotic therapy induces a rapid and extremely potent release of either endotoxin or cell wall fragments, it is recognized that decreasing injury during infection necessarily involves an effort to decrease the inflammation generated by antibiotic therapy itself. In this context, the ability to modulate leukocyte diapedesis, especially during antibiotic therapy of pulmonary infection, is currently of intense interest.

Recently, it has become clear that endotoxin and cell walls activate inflammatory mediators in mechanistically different ways.[5] This complicates the development of strategies aimed at down-modulating inflammation as a mechanism of reducing tissue injury. However, a common end-result of mediator release in both gram positive and gram negative infection is leukocyte diapedesis, a process which in turn is a key contributor to pulmonary damage. Inhibition of leukocyte trafficking is therefore not only a rational target for adjunct therapy to serious pneumonia, it is also one of the very few targets shared by gram positive and gram negative diseases.

Migration of leukocytes from blood into the alveolar space is a three-step process. The first step involves margination of the leukocyte by the interaction of its surface carbohydrates with selectins on the surfaces of the endothelial cells. Thus, circulating carbohydrates or selectin analogues competitively inhibit the ability of leukocytes to find inflamed endothelia. The second step is the activation of the rolling leukocyte by chemokines, particularly platelet activating factor (PAF). PAF plays a major role in pulmonary inflammation and PAF receptor antagonists are specifically useful in this context.[4] The third step is the actual transmigration event from blood to alveoles. The key molecules for this event are the CD18 family of leukocyte integrins. Antibodies to CD18 block leukocyte accumulation in lung when given intravenously. Similarly, molecules that bind receptors for CD18, such as ICAMs on the endothelium, also block leukocyte movement. However, there is one instance in which this is not the case. There appears to be a unique CD18-independent migration that accounts for 50% of the leukoctyes entering the lung during pneumococcal pneumonia.[4,6] Given the availability of three steps in leukocyte migration as potential anti-inflammatory targets, there exists a number of options for leukocyte-directed anti-inflammatory therapy. This approach is likely to be fruitful for the development of useful partner drugs for the amelioration of pulmonary damage during pneumonia as well as acute systemic states such as ARDS.

### References

1. Tuomanen E, Rich R, Zak O. Induction of pulmonary inflammation by components of the pneumococcal cell surface. *Am Rev Respir Dis* 1987; **135**: 869–874.
2. Tuomanen E, Pollack H, Parkinson A, *et al.* Microbiological and clinical significance of a new property of defective lysis in clinical strains of pneumococci. *J Infect Dis* 1988; **158**: 36–43.
3. Heiss L, Lancaster J, Corbett J, Goldman W. Epithelial autotoxicity of nitric oxide: role in the respiratory cytopathology of pertussis. *Proc Natl Acad Sci USA* 1994; **91**: 267–270.
4. Cabellos C, MacIntyre DE, Forrest M, Burroughs M, Prasad S, Tuomanen E. Differing roles of platelet-activating factor during inflammation of the lung and subarachnoid space. *J Clin Invest* 1992; **90**: 612–618.
5. Tuomanen E, Austrian R, Masure H. The pathogenesis of pneumococcal infection. *N Engl J Med* 1995; **332**: 1280–1284.
6. Doerschuk CM, Winn RK, Coxson HO, Harlan JM. CD18-dependent and -independent mechanisms of neutrophil emigration in the pulmonary and systemic microcirculation of rabbits. *J Immunol* 1990; **144**: 2327–2333.

### Serine proteinase genes in leukocytes

*R. A. Stockley and K. M. McConn*
Department of Medicine, Queen Elizabeth Hospital, Edgbaston, Birmingham, UK

Leukocytes contain several serine proteinases, including neutrophil elastase, proteinase 3 (Wegeners antigen) and Cathepsin G. These three enzymes have been implicated in the pathogenesis of chronic lung disease since both neutrophil elastase and Cathepsin G have also been shown to cause chronic bronchial disease and for this reason have been implicated in the pathogenesis of several chronic progressive lung diseases.

At present little is known about these proteinases and their regulation. The genes are distributed at different sites. Neutrophil elastase and proteinase 3 are found on chromosome 19, D13.3[1] whereas Cathepsin G is found on chromosome 14 Q 11.2.[2] These genes are expressed during differentiation of neutrophil in the bone marrow and currently no evidence exists to indicate that they are expressed in the fully differentiated cell. The best characterized gene, at present, is neutrophil elastase which is expressed at the promyelocyte stage during the differentiation of the neutrophil and its expression, therefore, is both tissue specific and time specific during the differentiation process.

The 5′ flanking region of the neutrophil elastase gene contains a CAAT box, TATA box and GC rich elements that are consistent with a functional promoter. Nucleotide deletion studies have suggested that other regulatory sequences are present within 200 base pairs upstream from the initiation codon. Han and colleagues[3] recognized both positive and negative regulatory sequences (–149 to –102 and –196 to –153 respectively) whereas Srikanth and Rado[4] identified a further positive regulatory sequence (–106 to –76). The sequences are active in promonocytic cells and not in other cell lines including the hep G2 or HELA cells, indicating that they may be tissue specific.

Studies with promonocytic and promyelocytic cell lines have shown that expression of the elastase gene can be decreased by factors that stimulate cell differentiation. In addition the use of anti-sense oligonucleotide probes, to proteinase gene transcripts in the HL60 cell line has actually been shown to induce differentiation towards a monocyte.[5] The implications of this latter finding is currently unknown.

The importance of the neutrophil elastase gene is indicated in several genetic defects. In animals, the beige mouse is a genetic variant in which the neutrophil elastase gene is not expressed. Neutrophils from these animals are therefore devoid of this serine proteinase. Infection studies have shown that the animals have normal neutrophil recruitment to the lungs but most importantly that their survival is much enhanced.[6] This suggests that in a heightened inflammatory state associated with infection, too much neutrophil elastase may be detrimental and would be consistent with our concepts of chronic infection in man.[7]

The human equivalent of the beige mouse is the Chediak Higashi syndrome. Mature neutrophils show abnormal empty granule formation. The cells have a chemotactic defect and do not contain neutrophil elastase.

Studies have shown that Cathepsin G is also absent in the mature neutrophils and confirmation has been obtained recently that proteinase 3 is also deficient (unpublished observations in collaboration with P. Hiemstra, Leiden). However, the studies of bone marrow have shown that immature neutrophils express both the elastase and Cathepsin G gene and that these enzymes are present prior to full maturation.[8] Since these serine proteinases are coded for on different parts of the human genome and since at least two of them are expressed in early differentiation in the bone marrow, it seems likely that the defect in Chediak Higashi syndrome is a common one that is involved in post-translational modification of the products. Studies have shown that all three enzymes are produced as a preproprotein. The amino terminal sequence is cleaved to provide the pro-enzyme and a final dipeptide is cleaved at a common site as indicated in the Fig. 1. Recent studies have suggested that the defective enzyme may be a cysteine dipeptidase but confirmation is awaited.

Studies in lung disease have shown that the amount of neutrophil elastase in leukocytes from patients with bronchiectasis is increased compared to age matched healthy controls and subjects with emphysema.[9] This increase in expression of enzyme is not unique since patients with rheumatoid arthritis and diabetes have also been shown to have inceased neutrophil elastase content of the PMN. The mechanisms involved in this excess enzyme production

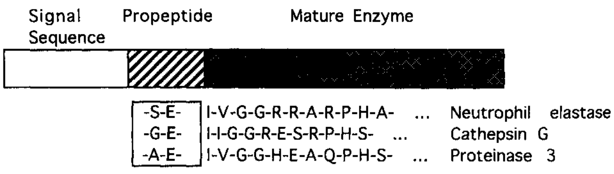

FIG. 1.

are unknown, but may clearly influence the subsequent tissue damage that occurs. The results would suggest that mechanisms exist for modulating the amount of neutrophil elastase within the PMN and this presumably occurs during differentiation. The reasons for increased neutrophil elastase content may relate to increased transcription, more efficient translation of the messenger RNA, modifications in intracellular processing and storage of transport to the cell membrane. Further studies of these processes may determine the exact mechanism but also provide potential therapeutic targets for the modulation of proteinase expression.

### References

1. Zimmer M, Medcalf RL, Fink TM, Mattmann C, Lichter P, Jenne DE. Three human elastase-like genes coordinately expressed in the myelomonocyte lineage are organized as a single genetic locus on 19 pter. *Proc Natl Acad Sci USA* 1992; **89**: 8215–8219.
2. Heusel JW, Hanson, RD, Silverman GA, Ley TJ. Structure and expression of a cluster of human haematopoietic serine protease genes found on chromosome 14q 11.2. *J Biol Chem* 1991; **266**: 6152–6158.
3. Han J, Unlap, T, Radion TA. Expression of the human neutrophil elastase gene: positive and negative transcriptional elements in the 5′ flanking region. *Biochem Biophys Res Comm* 1991; **181**: 1462–1468.
4. Srikanth S, Rado TA. A 30-base pair element is responsible for the myeloid-specific activity of the human neutrophil elastase promoter. *J Biol Chem* 1994; **269**: 32626–32633.
5. Bories D, Raynal M, Solomon DH, Darzynklewicz Z, Cayre YE. Down-regulation of serine proteinase myeloblastin causes growth arrest and differentiation of promyelocytic leukaemia cells. *Cell* 1989; **50**: 956–968.
6. Tanaka E, Yuba Y, Sato A, Kuze F. Effects of the beige mutation on respiratory tract infection with *Pseudomonas aeruginosa* in mice. *Exp Lung Res* 1994; **20**: 351–366.
7. Stockley RA. Bronchiectasis – new therapeutic approaches based on pathogenesis. In: *Clinics in Chest Medicine*, Vol. 8, No. 3. Philadelphia: W. B. Saunders, 1987: 481–494.
8. Burnett D, Ward, CJ, Stockley RA, *et al.* Neutrophil elastase and Cathepsin G protein in messenger RNA expression in bone marrow from a patient with Chediak higashi syndrome. *J Clin Path* 1995; **48**: M28–M34.
9. Burnett D, Chamba A, Hill SL, Stochley RA. Neutrophils from subjects with chronic obstructive lung disease show enhanced chemotaxis and extracellular proteolysis. *Lancet* 1987; **11**: 1043–1046.

### Proteinase inhibitor therapy

*Gerd Döring* and Gabriel Bellon***

*Hygiene Institut, Universität Tübingen, Germany and

**Hôpital Lyon-Sud, Lyon, France

There is a growing body of evidence that the consequences of neutrophil activation in the infected airways of patients with cystic fibrosis (CF) are at least partly responsible for respiratory failure and death in CF. Neutrophils release lysosomal metalloproteinases and serine proteinases upon activation and besides active collagenase and cathepsin G, high levels of elastase have been detected in bronchial lavage or sputum samples of CF patients.[1,2] Apparently, an elastase/anti-elastase imbalance is present in the inflamed CF airways and high amounts of the major endogenous elastase inhibitor, α1-proteinase inhibitor (α1-PI), are proteolytically cleaved and inactivated.[1] The other serine proteinase inhibitors, secretory leukocyte protease inhibitor (SLPI) and α2-macroglobulin seem to play a minor role in protecting the lower respiratory tract from neutrophil elastase.

Neutrophil elastase plays a major role in the pathophysiology of chronic inflammation in CF as well as in other disorders. Besides inactivation of α1-PI, it cleaves lung elastin and fibronectin, stimulates airway gland secretion, reduces the ciliary beat frequency and impairs opsonophagocytosis at the levels of immunoglobulins, complement and the complement receptor CRl on neutrophils.[1] These events may start very early in the life of a CF patient, since elevated levels of elastase have been found in CF patients younger than 6 months.[3,4] In older patients, they may be present not only in severely diseased patients but also in CF patients with stable, clinically mild lung disease.[5]

The proven elastase/anti-elastase imbalance suggests a strategy of increasing the antiproteinase levels in the lung by supplementation with suitable inhibitors.[1] A wide variety of inhibitors has been developed for neutrophil elastase, but only a few may be applied in humans. α1-PI is one of them. A trial with aerosolized α1-PI in a small number of CF patients for several weeks gave promising results:[3] when 12 patients received 1.5–3.0 mg/kg every 12 h for 1 week by aerosol, neutrophil elastase activity was suppressed in the epithelial lining fluid (ELF) and the anti-neutrophil elastase capacitiy was restored when ELF α1-PI reached 8 μM/l. Furthermore, killing of *Pseudomonas aeruginosa in vitro* was enhanced. However, in another ongoing trial in Lyon, in which the same dose of α1-PI was aerosolized twice daily for 3 days, similar results have not been obtained. When neutrophil elastase activity was assessed one day before and 8 days after aerosolization in bronchoalveolar lavage and daily in sputum samples no significant reduction of enzyme activity was reached. Positive results were obtained in a multicenter study of aerosolized α1-PI which completed 22 CF patients and where 100 mg, 200 mg or 350 mg were given every 12 h for 4 weeks. In all patients except one, elastase activity decreased and/or the capacity to inhibit added exogenous elastase increased in ELF. However, sputum elastase activities did not correlate well with ELF activities. It was concluded that biochemically effective levels of α1-PI (prolastin) can be safely delivered but further studies are needed for a better evaluation of the application of α1-PI in CF.

### References

1. Döring G, Knight R, Bellon G. Immunology of cystic fibrosis. In: Hodson ME, Geddes DM, eds. *Cystic Fibrosis*. London: Chapman and Hall, 1994; 99–129.
2. Goldstein W, Döring G. Lysosomal enzymes and proteinase inhibitors in the sputum of patients with cystic fibrosis. *Am Rev Respir Dis* 1986; **134**: 49–56.
3. Khan TZ, Wagener JS, Bost T, Martinez J, Accurso FJ, Riches DWH. Early pulmonary inflammation in infants with cystic fibrosis. *Am J Crit Care Med* 1995; **151**: 1075–1082.
4. Birrer P, McElvaney NG, Rüdeberg A, *et al.* Protease-antiprotease imbalance in the lungs of children with cystic fibrosis. *Am J Crit Care Med* 1994; **150**: 207–213.
5. Konstan MW, Hilliard KA, Norvell TM, Berger M. Bronchoalveolar lavage findings in cystic fibrosis patients with stable, clinically mild lung disease suggest ongoing infection and inflammation. *Am J Crit Care Med* 1994; **150**: 448–454.
6. McElvaney NG, Hubbard RC, Birrer P, *et al.* Aerosol α1-antitrypsin treatment for cystic fibrosis. *Lancet* 1991; **337**: 392–394.
7. Berger M, Konstan MW, Hilliard J. Aerosolized prolastin (α1-protease inhibitor) in CF. Abstr. presented at the *9th Annual North American CF Conference*, Dallas October 1995.

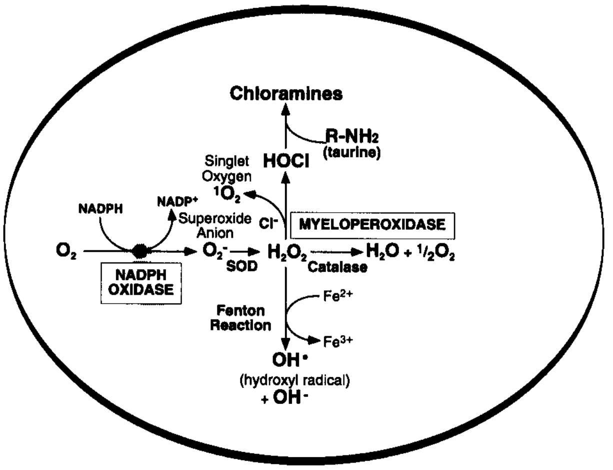

FIG. 1. Reactions leading to oxidant generation following neutrophil activation.

### Oxidative metabolism in polymorphonuclear neutrophils

*Véronique Witko-Sarsat and Béatrice Descamps-Latscha*

INSERM U25, Hôpital Necker, 161 rue de Sèvres 75015, Paris, France

In order to achieve its principal function which is the host defence against pathogens, neutrophil can use two different microbicidal systems.[1]

The non-oxygen dependent pathway consists of proteinases and antibiotic proteins stored in azurophil granules. Neutrophil elastase, which is a serine proteinase released upon neutrophil activation, has led to extensive studies in the context of cystic fibrosis (CF). The high levels of neutrophil elastase in CF airways has evidenced the importance of neutrophil-derived mediators in the inflammatory process in CF.

The second microbicidal system is the oxygen-dependent pathway leading to oxidant generation.[2] Unlike proteinases, no extensive studies on the oxidant-generating systems have been carried out in CF. However, a growing body of evidence has shown an imbalance between oxidants and antioxidants in CF, thus pointing out an oxidative stress.

Oxidative metabolism activation, known as the respiratory burst, first involves NADPH oxidase.[3] Activation requires cellular components from both the cytosol and the cell membrane. This enzymatic complex is an electron transport chain driven by an NADPH oxidase. The membrane proteins are composed of a cytochrome b558 which consists of a highly glycosylated large 91 kD subunit (gp91phox, glycoprotein 91 kD phagocyte oxidase) and which contains binding sites for NADPH and FAD and a small 22 kD protein subunit (p22 phox). The cytoplasmic subunits are p47phox (which binds to gp91phox after phosphorylation by protein kinase C), p67phox and a GTP-binding protein, Rac1 or Rac2, which hydrolyzes GTP in GDP and is involved in the dissociation of the active complex. After assembly, the NADPH complex is functional, resulting in the univalent reduction of molecular oxygen to superoxide anion. This superoxide is converted to hydrogen peroxide ($H_2O_2$) spontaneously or by the means of superoxide dismutase. In the presence of iron salts, superoxide anion and hydrogen peroxide may interact further to produce hydroxyl radicals (OH•) (see Fig. 1).

Myeloperoxidase is the second important enzyme in the oxidative metabolism of neutrophils. Myeloperoxidase is stored in azurophil granules and represents 5% of the neutrophil dry weight. In the presence of $H_2O_2$ and chlorine, it catalyses the formation of hypochlorous acid (HOCl).[4] The formation of singlet oxygen, a potent microbicidal agent, has also been described.[5] HOCl can react with endogenous amines ($R\text{-}NH_2$) to generate chloramines (RNH-Cl).[6] Taurine, a beta-amino acid considered as a HOCl scavenger and very abundant in neutrophils is the most common substrate used to form chloramines. The oxidants such as superoxide anion, $H_2O_2$ and even HOCl are very reactive and labile species whereas chloramines are stable enough to accumulate in the extracellular milieu; they have thus been called 'long-living oxidants'. The presence of long-living oxidants have been recently reported in sputum from CF patients.[7]

It becomes evident from several studies that superoxide anion or $H_2O_2$ may not be the microbicidal species and it is usually admitted that

HOCl, perhaps via the formation of singlet oxygen,[5] can be the most toxic substance.[8]

The exploration of neutrophil oxidative metabolism in CF has led to divergent results, because of the wide heterogeneity in the clinical state of the CF patients and because of the difference in the methodology.

It seems that defects in intracellular transduction found in CF epithelial cells were not present at the level of the neutrophil. In fact, increase in cellular cyclic AMP triggered by several pharmacological agents (adrenaline, theophyllin and forskolin) had the same inhibitory effect on neutrophil cellular functions such as superoxide anion production, degranulation, membrane depolarization in CF subject as in controls.[9] It has been shown that the luminol enhanced chemiluminescence which measured the intracellular MPO-dependent oxidant formation of neutrophils stimulated by opsonized zymosan had the same intensity in CF patients and in controls. However, the kinetics of the reaction were different: the time to reach the maximum was decreased in CF. Moreover, this parameter appears to be correlated with the clinical state of the patients. These authors thus concluded that neutrophils from CF patients were 'primed'.[10] Other studies on monocytes and neutrophils have shown an increase of luminol enhanced chemioluminescence following stimulation by f-MLP, PMA and A23187.[11]

Investigations on neutrophil oxidative metabolism are hampered by the fact that it is difficult to differentiate between a possible activation state due to recurrent infections of CF patients and a possible 'constitutive' defect which could be specific of CF. Investigation of neutrophils from CF heterozygote subjects, who are obligate carriers of the CFTR mutation and clinically asymptomatic, could provide a mean to discriminate between the two possibilities. It has been reported that monocytes from CF homozygotes and heterozygotes showed increased adherence on glass.[12] In a recent study carried out in our laboratory, we have shown a disturbance in MPO-dependent oxidant generation in neutrophils from CF homozygotes and heterozygotes as compared to controls. The mechanism underlying this hyperactivity seems to involve the sodium/proton exchange system as pharmacological blockers of this exchange system such as amiloride or EIPA or a sodium-deprived buffer corrected this hyperactivity (Witko-Sarsat *et al.*, submitted for publication).

In conclusion, the neutrophil plays a pivotal role in CF inflammation and remains the more potent in cellular oxidant-generating systems. It thus seems of special relevance to explore their mechanisms of formation, to study the consequence of their massive release as well as the clinical consequences.

### References

1. Witko-Sarsat V, Descamps-Latscha B. Phagocyte-derived oxidants and proteinases as immunomodulatory mediators in inflammation. *Mediat Inflamm* 1994; **3**: 257–273.
2. Babior BM. Oxygen-dependent microbial killing by phagocytes. *N Engl J Med* 1978; **298**: 659–668.
3. Gallin JI, Leto TL, Rotrosen D, Kwong CH, Malech HL. Delineation of the phagocyte NADPH oxidase through studies of chronic granulomatous diseases of childhood. *Curr Opin Immunol* 1991; **4**: 53–56.
4. Klebanoff SJ. Myeloperoxidase-halide-hydrogen peroxide antibacterial system. *J Bacteriol* 1969; **95**: 2131–3138.
5. Allen RL, Stjernholm R, Steele RH. Evidence for the generation of an electronic excitation state(s) in human polymorphonuclear leukocytes and its participation in bactericidal activity. *Biochem Biophys Res Commun* 1972, **47**: 679–684.
6. Weiss SJ, Lampert MB, Test ST. Long-lived oxidants generated by human neutrophils: characterization and bioactivity. *Science* 1983; **222**: 623–628.
7. Witko-Sarsat V, Delacourt C, Rabier D, Nguyen AT, Descamps-Latscha B. Presence of long-lived oxidants in sputum of cystic fibrosis patients. *Am J Resp Crit Care Med* 1995; **152**: 1910–1912.
8. Passo SA, Weiss SJ. Oxidative mechanisms utilized by human neutrophils to destroy *Escherichia coli*. *Blood* 1984; **3**: 1361–1368.
9. Suter SI, Chevallier KH, Krause Lew DP. Effect of cyclic adenosine monophosphate elevations on functional responses of polymorphonuclear leukocytes from patients with cystic fibrosis. *Pediatr Pulmonol* 1989; **6**: 237–241.
10. Roberts RL, Stiehm ER. Increased phagocytic cell chemiluminescence in patients with cystic fibrosis. *Am J Dis Child* 1989; **143**: 944–950.
11. Kemp T, Schram-Doumont A, Van Geffel R, Kram R, Szpirer C. Alteration of the N-formyl-methionyl-leucyl-phenylalanine-induced response in cystic fibrosis neutrophils. *Pediatr Res* 1986; **20**: 520–526.
12. Regelmann WE, Lunde LM, Porter PT Quie PG. Increased monocyte chemiluminescence in cystic fibrosis patients and in their parents. *Pediatr Res* 1986; **20**: 619–622.

### The role of oxidative stress in cystic fibrosis

*Frank J. Kelly*
Cardiovascular Research, The Rayne Institute,
St Thomas' Hospital, London, UK

Although the genetic defect responsible for cystic fibrosis (CF) is well characterized, the events leading to the gradual destruction of lung tissue and the consequent decrease in lung function remain unclear. In an attempt to improve this situation our Group has over recent years, addressed the specific role of oxidative stress and antioxidant defences in the pathogenesis of CF[1–5]. Two overriding features have characterized the findings from this series of studies. First, gross deficiencies of fat-soluble antioxidants such as α-tocopherol (vitamin E) have not been found. This presumably reflects the good clinical and nutritional management now provided in CF clinics.

Second and more disturbing, despite relatively normal antioxidant concentrations, many CF patients exhibit evidence of oxidative stress. Blood plasma obtained from CF patients has a decreased oxygen radical trapping capacity of plasma in CF patients, indicating an increased susceptibility to oxidative damage compared to control subjects.[1] Confirming this finding, we have demonstrated the presence of oxidized lipids (hydroperoxides) and proteins (carbonyls) in the plasma of CF patients, implying free radical-mediated tissue damage.[2] In addition, we have shown that the DNA oxidation product, 8-hydroxydeoxyguanosine is present in the urine of CF patients.[3] As already mentioned the interesting, and yet disturbing aspect of all these findings is that oxidative stress was detected in CF children whose circulating antioxidant defences (vitamin E, ascorbic acid, uric acid and total plasma sulphydryls) were within the normal age-related range. These findings, therefore, not only provide confirmation that increased radical production is a component of CF aetiology, but that normal concentrations of circulating antioxidants are not sufficient in many CF patients to offset the consequences of their increased oxidative burden.

*Oxidative stress and the decline in lung function in CF*

Establishing the presence of markers of oxidative injury in fluids such as plasma and urine has provided a biochemical basis for free radical damage in CF, but determining the clinical relevance of these findings is clearly more important. In this respect we recently found that the age-related decrease in lung function in CF children is associated with lower plasma $\alpha$-tocopherol, ascorbic acid and sulphydryl concentrations. Moreover, the age-related reduction in pulmonary function correlated with elevated plasma malondialdehyde in CF children.[4] In addition, patients with severe lung dysfunction ($FEV_1 < 50\%$ predicted) had higher plasma concentrations of lipid hydroperoxides than those with mild to moderate lung dysfunction ($FEV_1 > 50\%$ predicted). We believe that these findings warrant a closer examination of the association between lung function and oxidative stress in CF. To this end, studies are required which focus directly on the lung.

*Antioxidant defences and markers of oxidative stress in sputum*

Sputum arises in the lung and as such may provide a more suitable medium than plasma to examine for markers of oxidative stress and antioxidants. In a pilot study, conducted in 11 CF patients we have demonstrated that it is possible, with minor modifications to existing methods, to measure $\alpha$-tocopherol, uric acid, ascorbic acid and total sulphydryls in sputum. Indeed, the concentrations and composition of these antioxidants indicate that sputum is a reasonable surrogate for the lining fluids of the upper respiratory tract. Despite this wide array of extracellular antioxidants, evidence was obtained for both protein and lipid oxidation in sputum from CF patients. We have not yet had the opportunity to determine whether the markers of oxidative stress in sputum correlate with those present in plasma of the same patients. Neither have we addressed the association between antioxidant defences, markers of oxidative stress in sputum and lung function in CF patients.

*Antioxidant therapy for CF*

Two findings have led us to believe that antioxidant supplementation, to above control levels, may prove to be a useful therapeutic benefit in CF. First, markers of oxidative stress, which are not present in control age-matched children, are present in many CF patients, whose circulating antioxidant defences fall within the normal age-matched range. Second, CF patients with the highest plasma antioxidant concentrations exhibit the best pulmonary function. We hypothesize that CF patients endure cyclic periods of oxidative stress associated with chronic respiratory infection and that when this oxidative stress exceeds the patients pulmonary antioxidant defences oxidative-lung injury ensues.[5] This injury, in combination with that arising from the protease/antiprotease imbalance, contributes to the pulmonary pathology seen in CF patients and in time results in compromised lung function. Combating this oxidative stress with additional antioxidant therapy may help to reduce lung injury, and hence decrease the rate of loss of lung function.

### References

1. Langley SC, Brown RK, Kelly FJ. Reduced free radical-trapping capacity and altered plasma antioxidant status in cystic fibrosis. *Pediat Res* 1993; **33**: 247–250.
2. Brown RK, Kelly FJ. Evidence for increased oxidative damage in patients with cystic fibrosis. *Pediat Res* 1994; **36**: 487–493.
3. Brown RK, McBurney A, Lunec J, Kelly FJ. Oxidative damage to DNA in patients with cystic fibrosis *Free Rad Biol Med* 1995; **8**: 801–806.
4. Brown RK, Wyatt H, Price J, Kelly FJ. Increased oxidative damage and decreased pulmonary function in cystic fibrosis. *Eur Res J* 1996; **9**: 334–339.
5. Brown RK, Kelly FJ. Role of free radicals in the pathogenesis of cystic fibrosis. *Thorax* 1994; **49**: 738–742.

### Therapeutic strategies against oxygen radical-induced cell damage?

*Dieter Worlitzsch, Gunda Herberth and Gerd Doring*
Hygiene Institut, Universität Tübingen, Germany

In cystic fibrosis (CF), the most common autosomal recessive disorder of white populations, abnormal exocrine gland secretions lead to chronic endobronchial infections with opportunistic bacterial pathogens such as *Pseudomonas aeruginosa*. As a consequence, large numbers of neutrophils are recruited to the infected airways where cell activation and lysosomal enzyme release occur.[1] Neutrophils respond to stimulation with a burst of oxygen consumption and the production of reactive oxygen species such as superoxide anion, hydrogen peroxide, hydroxyl radical and possibly singlet oxygen. As with lysosomal proteinases, these species are not only released into the phagolysosome but also outside of the phagocyte where they may be toxic to the host.

It is not yet certain whether toxic oxygen metabolites produced by stimulated PMN also contribute to lung injury in CF, as has been suggested in other airway diseases.[2] However, indirect evidence may support this hypothesis; high sputum concentrations of extracellular myeloperoxidase (MPO), a PMN-derived enzyme which transforms hydrogen peroxide ($H_2O_2$) into highly reactive oxygen metabolites, have been detected in CF patients,[3–6] and lung function has been inversely correlated with MPO levels.[4–6] In addition, deficiency of the antioxidant glutathione[7] and increased lipid peroxidation[8] have been reported to occur in CF patients. The presence of endogenous scavengers such as superoxide dismutase (SOD) which dismutates superoxide anion to hydrogen peroxide, or catalase which changes hydrogen peroxide to water and oxygen, has not been investigated in CF sputum or bronchoalveolar lavage samples until now. The human catalase gene in airway epithelial cells is a 'house keeping gene' which cannot be augmented by hyperoxia.[9]

Nevertheless, the amount of tissue damage and degree of protein inactivation by oxidative attack in CF and other disorders has not been clearly defined. Of considerable interest is whether al-proteinase inhibitor (al-PI) undergoes oxidative inactivation. A number of studies have shown that $Met_{353}$, an important residue of the serine proteinase binding site, is particularly susceptible to oxidative attack, the result being a substantial reduction in the ability of the inhibitor to bind neutrophil elastase.[10]

Provided that further proof of the hypothesized oxidant/antioxidant imbalance in CF can be obtained, which possibilities do we have to protect the lung epithelium and biological important proteins, fatty acids, carbohydrate moieties and DNA from oxygen radical-induced damage? Several strategies have been developed including the application of rhSOD[11] or selenium,[12] aerosolization of N-acetylcysteine as a precursor of glutathione[13] and the augmentation of the antioxidant screen of the CF epithelial surface using replication-deficient recombinant adenoviral vectors[2] or liposomes[14] containing human catalase cDNA.[2]

### References

1. Döring G, Knight R, Bellon G. Immunology of cystic fibrosis. In: Hodson ME, Geddes MD, eds. *Cystic Fibrosis*. London: Chapman and Hall, 1994; 99–129.
2. Crystal RG. Oxidants and respiratory tract epithelial injury: pathogenesis and strategies for therapeutic intervention. *Am J Med* 1991; **91 (suppl3c)**: 39S–44S.
3. Goldstein W, Döring G. Lysosomal enzymes and proteinase inhibitors in the sputum of patients with cystic fibrosis. *Am Rev Respir Dis* 1986; **134**: 49–56.
4. Meyer KC, Zimmerman J. Neutrophil mediators. *Pseudomonas* and pulmonary dysfunction in cystic fibrosis. *J Lab Clin Med* 1993; **121**: 654–661.
5. Regelmann WE, Sieferman CM, Herron JM, Elliott GR, Glawson CC, Gray BH. Sputum peroxidase activity corrrelates with the severity of lung disease in cystic fibrosis. *Pediatr Pulmonol* 1995; **19**: 1–9.
6. Koller DY, Götz M, Eichler I, Urbanek R. Eosinophilic activation in cystic fibrosis. *Thorax* 1994; **49**: 496–499.
7. Roum JH, Buhl R, McElvany NG, Borok Z, Crystal RG. Systemic deficiency of glutathione in cystic fibrosis. *J Appl Physiol* 1993; **75**: 2419–2424.
8. Portal BC, Richard MJ, Faure HS, Hadjian AJ, Favier AE. Altered antioxidant status and increased lipid peroxidation in children with cystic fibrosis. *Am J Clin Nutr* 1995; **61**: 843–847.
9. Yoo JH, Erzurum SC, Hay JG, Lemarchand P, Crystal RG. Vulnerability of the human airway epithelium to hypoxia. Constitutive expression of the catalase gene in human bronchial epithelial cells despite oxidant stress. *J Clin Invest* 1994; **93**: 297–302.
10. Matheson NR, Wong PS, Travis J. Enzymatic inactivation of human alpha 1-proteinase inhibitor by neutrophil myeloperoxidase. *Biochem Biophys Res Commun* 1979; **88**: 402–409.
11. Koyama S, Kobayashi T, Kubo K, Sekiguchu M, Ueda G. Recombinant-human superoxide dismutase attenuates endotoxin-induced lung injury in awake sheep. *Am Rev Respir Dis* 1992; **145**: 1404–1409.
12. Sies H. Antioxidant activity in cells and organs. *Am Rev Respir Dis* 1987; **136**: 478–480.
13. Leff JA, Wilke CP, Hybertson BM, Shanley PF, Beehler CJ, Repine JE. Postinsult treatment with N-acetyl-L-cysteine decreases IL-1 induced neutrophil influx and lung leaks in rats. *Am J Physiol* 1993; **265**: L501–L506.
14. Thibeault DW, Rezaiekhaligh M, Mabry S. Prevention of chronic pulmonary oxygen toxicity in young rats with liposome-encapsulated catalase administrated intratracheally. *Pediatr Pulmonol* 1991; **11**: 318–327.

### Pro-inflammatory cytokines and their inhibitors

*Jean-Michel Dayer*
Division of Immunology and Allergy, University Hospital, 1211 Geneva 14, Switzerland

Simultaneously to the inflammatory process mediated by pro-inflammatory substances (cytokines such as Il-1 and TNFα), the organism resorts to natural mechanisms which limit the extension of the inflammation. Among them are: (1) specific cytokine antagonists at the receptor level (IL-1 Ra); (2) cytokines whose biological inhibitory functions oppose pro-inflammatory cytokines such as IL-1 and TNFα (e.g. IL-4, Il-13, Il-10, TGFβ); (3) inhibitors acting as binding proteins to the ligand (e.g. soluble receptors of cytokines) or plasma proteins acting as carriers; (4) natural enzyme inhibitors converting inactive procytokines into active ones; (5) enzymes cleaving cytokine membranous receptors; (6) decrease of cytokine receptor expression; (7) enzymes degrading proinflammatory cytokines; (8) natural autoantibodies to cytokines.

Inhibition can also be contrived by interfering at the following levels: (1) the stimulus inducing the production of proinflammatory molecules (e.g. anti-CD14) in relation to LPS stimulation; anti-CD11 and anti-CD69 in relation to cell–cell interaction resulting in the production of IL-1, TNFα, PGE2 and metalloprotease; (2) the transduction, transcription and translational processes; (3) the maturation and secretion of cytokines; (4) the functional structure of cytokines (muteins).

Factors to be considered when examining the concept of cytokine/cytokine inhibitors are: (1) the chronology of appearance of the molecules; (2) their relative levels are more important than absolute levels; (3) the potentially beneficial effect, for a short period, of a proinflammatory molecule in host defense; (4) the complexity of some cytokine systems such as the IL-1 system which prossesses at least two types of natural antagonists (Il-1Ra, Il-1SR) leading to the paradox of enhanced pro-inflammatory activity; (5) the formation of unstable cytokine/cytokine inhibitor complexes.

Therefore, the concept of cytokine inhibitors may not be considered an 'on/off' but a 'buffer' system. Another important aspect of the potential impact of cytokine inhibitors is the dissociation between their immunosuppressive and anti-inflammatory action. TNF-R: Fc fusion protein, for instance, is hundred times more potent, in inhibiting metalloproteases and PGE2 production by fibroblasts than in inhibiting T cell proliferation and cytokine production on T cells. It is therefore important in devising therapeutical approaches in the future to pinpoint inhibitors of cytokines that are relevant primarily in immune-inflammatory or destructive events.

### References

1. Arend WP, Dayer JM. Inhibition of the production and effects of IL-1 and TNFα in rheumatoid arthritis. *Arthritis Rheum* 1995; **38**: 151–160.
2. Burger D, Dayer JM. Inhibitory cytokines and cytokine inhibitors. *Neurol* 1995; **45 (suppl.6)**: S39–S43.
3. Nicod LP, Galve de Rochemonteix B, Dayer JM. Modulation of Il-1 receptor antagonist and TNF-soluble receptors produced by alveolar macrophages and blood monocytes. In: *Annals of the New York Aademy of Sciences*, Vol. **725**, 1994, pp. 323–330.
4. Dayer JM, Arend WP. Cytokines and growth factors. In: Kelley WN, Harris ED, Ruddy S, Sledge CB, eds. *Textbook of Rheumatology*, 5th edn. Philadelphia: Saunders (in press).
5. Isler P, Vey E, Zhang JH, Dayer JM. Cell surface glycoproteins expressed on activated human T cells induce production of interleukin-1 beta by monocytic cells: a possible role of CD69. *Eur Cytokine Netw* 1993; **4**: 15–23.
6. Lacraz S, Isler P, Vey E, Welgus HG, Dayer JM. Direct contact between T lymphocytes and monocytes is a major pathway for induction of metalloproteinase expression. *J Biol Chem* 1994; **269**: 22027–22033.
7. Burger D, Chicheportiche R, Giri J, Dayer JM. The inhibitory activity of human interleukin-1 receptor antagonist is enhanced by type II interleukin-1 soluble receptor and hindered by type I interleukin-1 soluble receptor. *J Clin Invest* 1995; **96**: 38–41.
8. Nicod LP, El Habre F, Dayer JM, Boerhinger N. Il-10 decreases tumor necrosis factor α and β in alloreactions induced by human lung dendritic cells and macrophages. *Am J Respir Cell Mol Biol* 1995; **13**: 83–90.
9. Lacraz S, Nicod R, Welgus HG, Dayer JM. IL-10 inhibits metalloproteinase and stimulates TIMP-1 production in human mononuclear phagocytes. *J Clin Invest* 1995; **96**: 2304–2310.

### Cytokines in cystic fibrosis lung disease

*Melvin Berger, Tracy Bonfield, James Panuska and Michael Konstan*

Departments of Pediatrics and Medicine, Case Western Reserve University School of Medicine, Cleveland, Ohio 44106, USA

Several characteristics of CF, including the excessive influx of neutrophils into the lungs, cachexia and hyperglobulinaemia, suggest the involvement of pro-inflammatory cytokines such as interleukin (IL)-1, tumour necrosis factor (TNF-α), IL-8 and IL-6. Because local synthesis and actions of cytokines are often more important than systemic concentrations and/or effects, we have focused on measuring cytokine concentrations and determining their sites of production within the lung itself, using bronchoalveolar lavage (BAL) and airway brushing specimens. Concentrations of the pro-inflammatory cytokines IL-l, IL-6 and TNF-α are all dramatically elevated in BAL fluids from CF patients as compared to healthy controls. While no IL-8 is found in the BAL fluid of healthy controls, CF patients' fluids contained greater than 30 ng/ml of this potent neutrophil attractant, even when they were stable and remote from exacerbation. CF patients' BAL fluids contained modest elevations of the regulatory factors IL-l receptor antagonist and TNF-α soluble receptor fragments but the ratios of antagonist to cytokine were greatly reduced in the CF patients. IL-10, a regulatory cytokine, which suppresses production of the pro-inflammatory cytokines, was absent or markedly reduced in the CF specimens but present at 3.5 ng/ml in specimens from healthy controls. All of these differences were more pronounced in CF patients infected with *Pseudomonas* as compared with patients infected with *Haemophilus influenzae* and/or *Staphyloccocus aureus*, but similar changes were present in the latter patients as well. In one case of an infant in which intensive antibiotic treatment was able to eradicate detectable infection, the concentrations of IL-6 and IL-8 fell after treatment but were still markedly elevated as compared with controls. No IL-10 was detectable before or after antibiotic treatment.

To understand this imbalance towards pro-inflammatory cytokines, we evaluated the possible cellular sources of these cytokines in the CF lung. Fresh macrophages in CF BAL specimens, fixed and stained immediately after they were obtained from the lung, showed positive immunofluorescence for IL-1, IL-6, IL-8 and TNF-α and resembled healthy controls' macrophages which had been incubated with lipopolysaccharide *in vitro* before staining. In contrast, fresh macrophages from the controls which were not incubated *in vitro* were predominately negative. Interestingly, fresh macrophages from the CF patients stained positively for IL-10. This was also observed in LPS-treated macrophages from healthy controls but not in untreated controls' cells. Further studies, aimed at determining the source of IL-10 in the healthy controls' lavage fluid, showed that fresh bronchial epithelial cells obtained by brushing had positive immunofluorescence for IL-10, contained IL-10 mRNA, and secreted functionally active IL-10 when placed in primary culture *in vitro*. CF patients' bronchial epithelial cells contained much less IL-10 by immunofluorescence. These findings suggest that in the normal lung, the bronchial epithelial cells produce the anti-inflammatory cytokine IL-10 to down-regulate local inflammatory responses and prevent epithelial damage, whereas this suppressive mechanism is lost in the infected lung in CF. In contrast, in CF the bronchial epithelial cells are actively producing IL-6 and IL-8, which further exacerbate the inflammatory process.

Taken together, these observations suggest that the bronchial epithelial cells may play a major role in regulating local immunologic and inflammatory processes in the lung. This hypothesis, in turn, raises the questions of whether defective CFTR function could alter epithelial cell cytokine production, even in the absence of infection; and/or whether the responses of CF epithelial cells to stimuli that could upregulate IL-8 production and/or downregulate IL-10 production differ from the responses of normal cells.

### References

1. Bonfield TL, Panuska JR, Konstan MW, *et al.* Inflammatory cytokines in cystic fibrosis lungs. *Am J Respir Crit Care Med* 1995; **152**: 2111–2118.
2. Bonfield TL, Konstan MW, Burfiend P, Panuska JR, Hilliard JB, Berger M. Normal bronchial epithelial cells constitutively produce the anti-inflammatory cytokine interleukin-10, which is downregulated in CF. *Am J Respir Cell Mol Biol* 1995; **13**: 257–261.

### CFTR gene regulation by cytokines and anti-inflammatory drugs

*Aleksander Edelman, Françoise Besançon* and Marie-Yvonne Baudouin-Legros*
INSERM U323, CHU Necker and *INSERM U417, CHU St Antoine, Paris, France

Cystic fibrosis results from mutations in a 250 kb gene encoding the CFTR protein, a cyclic AMP-regulated $Cl^-$ channel. CFTR protein is located in the apical membranes of secretory epithelial cells.[1,2] Mutations in the gene result in the altered synthesis, processing or function of the protein. The dysfunction of this channel is, at least partly, responsible for defective fluid secretion in CF patients.[3] The disease has pleiotropic manifestations dominated by abnormalities of the airway epithelium surface which hypersecretes a thick mucus favouring inflammation and chronic infections.

Inflammation triggers the formation of a large number of both intracellular and tissular mediators able to affect the cell functions. For example, interferons (IFN), a family of multifunctional proteins produced in response to viral and bacterial infections, are major mediators of the host defence against the infectious agents.[4] IFNα and IFNβ are mainly synthesized by lymphocytes and fibroblasts, respectively. IFNγ, a key cytokine of the immune network, is synthesized by T lymphocytes and natural killer cells after antigen stimulation, and its crucial role in the defence against parasitic and viral infections was recently highlighted.[5] Another mediator of inflammation, the tumour necrosis factor α, TNFα, is synthesized and released by macrophages/monocytes as well as by B and T cells, Kupffer cells, glial cells and adipocytes,[6] and provokes pleitropic intracellular effects. IFNγ and TNFα also alter transepithelial ion transport but the mechanisms responsible for this action are not well defined.

Both the bacterial colonization and the tissular mediators of inflammation may affect the cell functions. However, whether they influence directly or indirectly CFTR function was not clearly established. This prompted the investigation of the effects of IFNs and of TNFα on CFTR gene expression.

Because inflammation and infection constitute the practical problem in cystic fibrosis, antibiotics and anti-inflammatory drugs are systematically administrated to treat CF patients. The present state of the art about these molecules suggests that they might affect the epithelial cell functions. Thus, we investigated the actions exerted by two different anti-inflammatory drugs (aspirin and dexamethasone) on CFTR gene expression.

Two adenocarcinoma cell lines (HT29 and T84 cells) known to express large amounts of CFTR protein served as experimental models. We found that IFNγ, at low concentrations (from 0.1 up to 100 IU/ml), down-regulated CFTR gene expression at mRNA and protein levels through a dose-dependent post-transcriptional mechanism. The effect was time-dependent: the IFNγ-induced mRNA decrease was significant at 3 hours and maximal after a 12 hour-incubation. INFα and β were inactive on CFTR gene expression in T84 and HT29 cells, suggesting that the effect of IFNγ was specific. The IFNγ-induced inhibition of CFTR gene expression evidenced at mRNA and protein levels was also found at the functional level: treatment with IFNγ reduced the cAMP-dependent $Cl^-$ fluxes. In HT29 cells, TNFα, which is concomitantly produced with IFNγ during the inflammatory and immune reactions, also reduced CFTR gene expression, and acted synergistically with IFNγ.[7]

These results suggest that the inflammatory processes may enhance the CF symptomatology and impair the efficacity of a gene therapy. Thus, to prevent the negative action of the two cytokines, we are investigating the effects of two molecules, representative of the distinct families of anti-inflammatory drugs, i.e., dexamethasone as an example of steroid-like molecules and aspirin as a non-steroid molecule, on CFTR gene expression. Under experimental conditions used, dexamethasone (1 μmol) does not exert any effect, either at mRNA or at a functional level. On the contrary, 48 hour-treatment of the T84 cells either with aspirin or with salicylic acid (0.5 to 1 mM) decreased the amount of CFTR transcripts and the cAMP-dependent Cl fluxes. The aspirin effect does not require *de novo* protein synthesis, since it subsists after treatment of the cells with cycloheximide. This effect is not due to a primer effect on adenylate cyclase since the forskolin-induced cAMP production is not modified by the treatment of the cells with aspirin (or salicylic acid).

These data suggest that production of IFNγ and TNFα in response to infections might worsen lung dysfunction in CF patients, and create a localized CF phenotype in non-CF patients with chronic bacterial or inflammatory lung disorders. Furthermore, our results also point out new action of aspirin commonly used as an anti-inflammatory drug in CF. High doses of aspirin, when applied chronically, might also diminish CFTR-related $Cl^-$ secretion and worsen status of the patient.

### References

1. Quinton P. Cystic fibrosis: a disease in electrolyte transport. *FASEB J* 1990; **4**: 2709.
2. Fuller C, Benos DJ. CFTR! *Am J Physiol* 1992; **263**: C267.
3. Welsh JM, Smith AE. Molecular mechanisms of CFTR chloride channel dysfunction in cystic fibrosis. *Cell* 1993; **73**: 1251.
4. De Maeyer E, De Maeyer-Guignard J. In: Sen GC, Lengyel P, eds. *Interferons and Other Regulatory Cytokines*. New York: John Wiley, 1988.
5. Dalton DK, *et al.* Multiple defects of immune cell function in mice with disrupted interferon-γ genes. *Science* 1993; **259**: 1739.
6. Lewis SA, *et al.* Modulation of epithelial permeability by extracellular macromolecules. *Physiol Rev* 1995; **75**: 561.
7. Besançon F, *et al.* Interferon-γ downregulates CFTR gene expression in epithelial cells. *Gastroenterology* 1995; **109**: 639.

### Anti-inflammatory therapy for cystic fibrosis: rational and prospects

*Michael W. Konstan and Melvin Berger*
Department of Pediatrics, Case Western Reserve University School of Medicine, Cleveland, Ohio 44106, USA

Many lines of evidence suggest that the accumulation of neutrophils into the airways is excessive and plays a major role in the lung destruction that continues to be the primary cause of morbidity and mortality in CF.[1] In addition to the destructive effects, there are many ways in which neutrophil products cause dysfunction of the lung and its defence mechanisms. These have been amply reviewed by other speakers at this symposium. Several recent studies[2,3] including our own work on patients with relatively mild lung disease,[4] make it clear that this excessive inflammatory process begins very early during the course of CF and continues unabatedly, although its intensity does vary from time to time.[5] Chronic infection in the lung undoubtedly is a major stimulus for this inflammatory response, and exacerbations of infection clearly lead to increased numbers of neutrophils and their damaging products. Some recent observations suggest that inflammation may precede infection[2,3] and/or that regulation of inflammation in the CF lung may be inherently abnormal, but the role of infectious exacerbations in increasing the inflammatory damage cannot be denied.

Clearly, treatment designed to decrease the inflammatory damage must include efforts to prevent and/or decrease the amount of infection. However, once *Pseudomonas* becomes firmly established, infection is rarely eradicated despite continuing evolution of new antibiotics. Bronchodilators, airway clearance techniques and DNase also contribute to the goal of preserving lung function, but despite all these efforts, the inflammatory process continues and inexorably worsens. For these reasons, additional strategies, such as anti-inflammatory agents that attempt to compensate for the basic defect (i.e., by the use of amiloride and/or UTP) and gene therapy are all being developed.

In contemplating anti-inflammatory therapy, it is obvious that the main target must be the neutrophil itself. The initial step in migration of neutrophils into the lung involves activation and attraction of the cells by soluble mediators. Many studies have documented that CF lung secretions contain high concentrations of all of the major classes of neutrophil chemo-attractants, most notably, $LTB_4$, C5a and IL-8 (reviewed in 1). It is therefore unlikely that antagonists of the production or receptor for any single one of these would have a major effect. Pharmacologic agents which might interfere with the adhesion molecules employed by the neutrophil to crawl out of the small blood vessels and into the epithelium have not yet reached clinical trials. Thus, the most practical approach is to interfere directly with neutrophil migration and function.

Because of the likely side effects of corticosteroids, which subsequently proved problematic in a large clinical trial in the US,[6] we investigated the use of non-steroidal anti-inflammatory drugs (NSAIDs), in particular, ibuprofen. Ibuprofen has direct effects in decreasing neutrophil migration and degranulation and at high but achievable concentrations, blocks the release of $LTB_4$ as well as inhibiting cyclo-oxygenase (reviewed in 7). Using the delivery of neutrophils to the alveolar crevices of the gingiva in the mouth as a model for the intrabronchial infection in the lung, we showed that ibuprofen interfered with movement of neutrophils *in vivo* in normal volunteers as well as in CF patients.[8] Most importantly, we found that administration of ibuprofen to animals with the agar bead model of chronic *Pseudomonas* infection reduced the area of inflammation in the lung without increasing the burden of infectious organisms.[7] After these preliminary studies, and once we had defined the pharmacokinetics of ibuprofen in patients with CF, we initiated a four-year clinical trial of this drug in patients with mild lung disease. Compared with placebo, ibuprofen-treated patients had significantly less decline in pulmonary function and Brasfield chest X-ray scores, preserved their percentage of ideal body weight, and tended to have fewer hospital admissions.[9] The effect was most pronounced in the youngest age group (5–13 years): the annual rate of decline in the percent predicted FEV1 was 88% slower for ibuprofen-treated patients compared to placebo. There were no significant adverse effects. Clinical use of ibuprofen is now being implemented throughout the US and other parts of the world. Establishing the appropriate dose by pharmacokinetic studies in each patient is important in individualizing therapy. Other NSAID trials have also been initiated and preliminary results are promising as well (reviewed in 10). Reports showing that inflammation is found even in infants with CF and our results which showed that the most dramatic effects of ibuprofen occurred in the youngest patients, suggest that anti-inflammatory therapy should be initiated very early in life. Until the potential of gene therapy is realized, anti-inflammatory agents are likely to be an increasingly important part of the therapeutic armamentarium in CF.

### References

1. Konstan MW, Berger M. Infection and inflammation in the lung in cystic fibrosis. In: PB Davis, ed. *Cystic Fibrosis*. New York: Marcel Dekker, 1993.
2. Kahn TZ, Wagener JS, Bost T, Martinez J, Accurso FJ, Riches DW. Early pulmonary inflammation in infants with cystic fibrosis. *Am J Respir Crit Care Med* 1995; **151**: 1075–1082.
3. Armstrong DS, Grimwood K, Carzino R, Carlin JB, Olinsky A, Phelan PD. Lower respiratory infection and inflammation in infants with newly diagnosed cystic fibrosis. *BMJ* 1995; **310**: 1571–1572.
4. Konstan MW, Hilliard KA, Norvell TM, Bergen M. Bronchoalveolar lavage findings in cystic fibrosis patients with stable, clinically mild lung disease suggest ongoing infection and inflammation. *Am J Respir Crit Care Med* 1994; **150**: 448–454.
5. Cantin A. Cystic fibrosis lung inflammation: early, sustained and severe. *Am J Respir Crit Care Med* 1994; **150**: 448–454.
6. Eigen H, Rosenstein BJ, FitzSimmons S, Schidlow DV. A multicenter study of alternate-day prednisone therapy in patients with cystic fibrosis; Cystic Fibrosis Foundation Prednisone Trial Group. *J Pediatr* 1995; **126**: 515–523.
7. Konstan MW, Vargo KM, Davis PB. Ibuprofen attenuates the inflammatory response to *Pseudomonas aeruginosa* in a rat model of chronic pulmonary infection: implications for anti-inflammatory therapy in cystic fibrosis. *Am Rev Respir Dis* 1990; **141**: 186–192.
8. Konstan MW, Hilliard KA, Davis PB. Effect of ibuprofen on neutrophil delivery to mucosal surcaces; *Pediatr Pulmonol Suppl* 1989; **4**: 152–153.
9. Konstan MW, Byard PJ, Hoppel CL, Davis PB. Effect of high-dose ibuprofen in patients with cystic fibrosis. *N Engl J Med* 1995; **332**: 848–854.
10. Konstan MW. Anti-inflammatory therapy in cystic fibrosis. In: *New Insights into Cystic Fibrosis*. New Jersey: Gardiner Caldwell SynerMer, Vol 3 (2), 1995.

### Different approaches to gene therapy for cystic fibrosis

*Patricia Lemarchand*

INSERM U25, Faculté de Médecine Necker-Enfants Malades, 75015 Paris, France

Gene therapy for cystic fibrosis (CF) has been first developed to replace *in vivo* the CF defective gene. Rapid progress towards gene transfer has been made since the identification of the CF gene, and the initial complementation experiments provided encouragement to investigators in search of gene transfer vectors to deliver the CF gene to airways. The ideal vector would efficiently deliver the gene to the appropriate target cells without causing toxicity or inflammation. Although none of the available vectors meets these criteria, studies in a number of animal models demonstrated gene transfer to the lung, and human trials have been initiated using recombinant adenoviruses and liposomes as vectors for gene transfer to airway epithelium. However, the underlying pathophysiology of CF remains unclear, and several issues have come up for successful CF gene therapy. First, at the cellular level, there is abundant evidence that CFTR is important for several cellular functions. Although the clinically relevant respiratory manifestations of CF are in the airways, it is not possible to evaluate CFTR function reproducibly in the human bronchial epithelium *in vivo*, and thus it is difficult to demonstrate at the site of the major clinical manifestations of the disease that a gene therapy strategy in humans actually corrects the functional deficiencies (including $Cl^-$ conductance and hyperabsorption of $Na^+$) associated with mutations in the CFTR gene. Second, at the organ level, CFTR has been localized to both the surface epithelium and submucosal glands in the lungs. None of the vectors being used in human trials efficiently achieves gene transfer to submucosal glands. Thus, if CFTR function in submucosal glands is necessary for normal airway clearance, current approaches are not likely to impact on disease pathogenesis in the cartilagenous airways. Finally, the relative sparing of pulmonary disease in CF patients who carry an allele encoding a partially defective CFTR molecule suggests that less than complete genetic reconstitution will be necessary for therapeutic efficiency. However, *in vivo* adenovirus-mediated gene transfer to the airway epithelia of nonhuman primates tends to be of low efficiency and patchy.

In summary, several key issues will need to be addressed for successful CF gene therapy. Besides CF gene transfer, 'non-specific' gene therapy strategies which may be applied to CF patients are being developed, including protection against oxidant stress and improvement of lung graft survival.[1-3]

*Anti-oxidative gene therapy for CF*

A major part of the airway damage in CF is mediated by oxidants released by inflammatory cells on the airway epithelial surface, particularly neutrophils. Because the number of neutrophils in the epithelial lining fluid (ELF) of individuals with CF are 100- to 1000-fold greater than in normal subjects, the potential burden for oxidants on the airway epithelium is enormous. The oxidant-antioxidant imbalance is placed further in favour of the oxidants in that respiratory ELF levels of glutathione are markedly reduced in CF compared with that in normal individuals. A strategy to protect the epithelium is to augment the anti-oxidant screen of the epithelial surface, thus allowing the inflammatory cells on the epithelial surface to phagocytize and kill bacteria, yet protecting the epithelium from damage by oxidants released by the phagocytes during this process. Preliminary *in vitro* studies have demonstrated that adenovirus mediated gene transfer of the human catalase gene could protect human epithelial cells against oxidant stress.

*Improvement of lung graft survival by gene therapy*

Lung transplantation has rapidly evolved into an effective therapy for end-stage pulmonary failure caused by CF. However, lung transplantation is associated with complications such as reperfusion injury and graft rejection. Pulmonary oedema with acute respiratory failure occurs in about 20% of patients after lung transplantation and is associated with increased early mortality. This acute respiratory failure results from a complex lung injury with both ischaemic and reperfusion components. Gene therapy targeted to the graft offers a promising approach to the prevention of these complications, by the transfer of antioxidant genes such as catalase and/or superoxide dismutase genes. As adenovirus vectors can transfer genes *in vivo* to the lung vasculature, we evaluated the feasibility of gene transfer to the lung graft in a porcine model of left lung allotransplantation, using an adenovirus vector containing a reporter gene. Adenovirus-mediated gene transfer to the lung graft was feasible *ex vivo*, but several parameters limited gene transfer efficiency and need to be improved before clinical application is attempted.

**References**

1. Alton EWFW, Geddes DM. Gene therapy for cystic fibrosis: a clinical perspective. *Gene Therapy* 1995; **2**: 88–95.
2. Wilson JM. Gene therapy for cystic fibrosis: challenges and futures directions. *J Clin Invest* 1995; **96**: 2547–2554.
3. Rosenfeld MA, Collins FS. Gene therapy for cystic fibrosis. *Chest* 1996; **109**: 241–252.

### Gene therapy in cystic fibrosis

*Gabriel Bellon and Andrea Pavirani**

Hospices Civils de Lyon, Unité "Pneumologie Pédiatrique-Mucoviscidose", Centre Hospitalier Lyon Sud, 69310 Pierre Bénite and *Transgène SA, 11 rue de Molsheim, 67082 Strasbourg, France

At present it is conceivable to think that gene therapy represents a way to treat or even prevent the respiratory manifestations of cystic fibrosis. Consistent with such a concept, there is sufficient evidence that Ad-CFTR, a recombinant replication-deficient adenovirus expressing the human cystic fibrosis transmembrane conductance regulator cDNA, can vectorize the expression of a functional CFTR (cystic fibrosis transmembrane conductance regulator) to the nasal and airway epithelia.

The clinical protocol was designed to assess the safety of single escalating doses of a replication defective adenovirus expressing the cystic fibrosis transmembrane conductance regulator gene (Ad-CFTR) when administered to the tracheobronchial portion of the airways and whether biological efficacy of CFTR delivery could be demonstrated.

Six cystic fibrosis patients received nasal instillation and subsequent aerosol (Optineb®, Air Liquide, Paris, France) administration of Ad-CFTR the following day. Doses (*pfu*) applied to the nose were $10^5$ (patients SG and PB), $10^7$ (patients FP and EP) and $4 \times 10^8$ (patients DS and FG), while aerosolized doses were $10^7$ (patients SG and PB), $10^8$ (patients FP and EP) and $5.4 \times 10^8$ (patients DS and FG), respectively. No acute toxic effects, no increase in the titer of anti-adenovirus antibodies and no spreading or shedding of Ad-CFTR were detected. In one patient Ad-CFTR DNA was found in the urine and blood two days after aerosolization. Ad-CFTR DNA was detected in nasal and bronchial brush samples, in BAL, in saliva and tonsils 21, 8, 14 and 4 days post virus administration, respectively. Ad-CFTR mRNA (RT-PCR on bronchial cells) and CFTR protein (immunochemistry on nasal and bronchial cells) were detected up to 14 days following Ad-CFTR administration.

These results show that the nebulization of Ad-CFTR is a possible approach for treating the respiratory manifestation of cystic fibrosis.

ACKNOWLEDGEMENTS. This work was supported by: Hospices Civils de Lyon, Ministère de la Santé, AFLM, Ministère de la Recherche, DRASS Rhône-Alpes, and Association Expector.

### Pulmonary inflammation consecutive to gene transfer

*Claire Danel*

Service d'Anatomie Pathologique, Hopital Laënnec, Paris, France

To accomplish gene therapy successfully, genetic information must be transferred and expressed in the target cells. Various vectors such as retroviruses, adenoviruses, adeno-associated viruses, liposomes and molecular conjugates have been developed to enhance this process. Adenovirus-based vectors have a number of properties that make them attractive vehicles for human gene therapy.[5,7] Their ability to transfer genetic material efficiently into lung epithelial cells has led them to be chosen for the first trials of human gene therapy for cystic fibrosis (CF).[2,3,5,7,8,10,11] The success of this approach will depend not only on the level and duration of transgene expression but also on its safety. Important questions remain regarding the clinical toxicity of administration of adenoviral vectors to the CF lung. In theory, several problems may occur following the use of recombinant adenoviruses, including the induction of cellular immunity, recombination to produce a replication competent virus, production of neutralizing antibodies reducing the efficacy of further application and inflammation at the site of therapy, with potential systemic consequences. Animal studies have addressed some of these important safety issues, especially in rodents and non-human primates. These studies are critical for assessing the potential toxicity associated with direct instillation of recombinant adenoviruses, such as the adenovirus containing human CFTR (AdCFTR), on airways and lung parenchyma.

*Pulmonary inflammation in animal species*

The cotton rat provides a good model for safety evaluations of human adenovirus-derived vectors.[6] In this animal model, with regard to the expression of the transfected gene in targeted cells, all cell types in the respiratory airway epithelium expressed the gene, with a good distribution of the vector throughout the respiratory tract as far as the terminal bronchioles but with relatively little gene expression in alveolar epithelium.[5,13] After *in vivo* intra-pulmonary administration of adenoviral vectors, animals displayed normal behaviour, and no morbidity or mortality was observed within a wide range of dosages.[13] In contrast it has been shown a mild to moderate dose-dependent transient pulmonary inflammation characterized by cellular infiltrates which disapeared in a few weeks.[2,3,6,13] The nature of the infiltrate changed from neutrophil-dominated during the first few days to one dominated by mononuclear cells such as macrophages and lymphocytes by day 10. No parenchymal, bronchial or bronchiolar epithelial cell damage or viral inclusions such as those observed after the administration of the wild-type adenovirus were observed.[3,6,13] Furthermore, in animals evaluated histologically for pulmonary fibrosis 28 days after vector administration, no increase in collagen fibres or architectural disorders were observed in the peribronchiolar, perivascular, alveolar or pleural areas.[13]

In non-human primates, baboons or rhesus monkeys, the major adverse effect associated with intrabronchial instillation of recombinant adenoviruses was pulmonary alveolar inflammation. The severity and location of the inflammatory infiltrate depended upon the concentration of the virus to which the lung tissue was exposed, and the inflammation was not restricted solely to the area where the virus was directly infused. Occurrence of inflammation outside the targeted regions is likely due to spillover during virus infusions.[9] Post-mortem microscopic analysis of the lung parenchyma demonstrated a dose dependent increase in inflammatory cells, primarily lymphocytes in the area where AdCFTR was delivered and which persisted for at least 2 months in some animals.[1] This inflammatory reaction appeared to develop over 2–3 weeks following gene administration. It started with mild to moderate perivascular accumulation of lymphocytes, and extended to the lung interstitium in animals receiving the highest dose ($10^9$ and $10^{10}$ pfu). Clinical tests did not accurately reflect the presence of lung inflammation, with the exception of alveolar infiltrates on chest radiographs and an increase in lymphocytes in bronchoalveolar lavage (BAL).[1,9] Abnormalities that were seen on chest radiographs resolved completely on follow-up examinations. The animals' physical appearances and behaviour appeared normal throughout the studies. In all these species, cotton rats or non human primates, examination of non-respiratory organs failed to show any abnormalities attributed to the transfection.

*Mechanisms that contribute to the inflammatory responses*

The precise mechanism of the lung inflammation observed in animals after instillation of recombinant adenovirus is unknown. Similar histopathologic appearances after administration of different types of replication-deficient adenovirus such as AdRSVBgal (containing the *Escherichia coli* lac Z) or Ad CFTR (containing the normal human CFTR cDNA) indicate that the inflammatory response is probably related to adenovirus components rather than to the transfected gene. It is unlikely that cell lysis from viral replication initiated the injury. No viral inclusions were seen and no recombinant or wild type viruses were detected by culture. The more likely cause to the pulmonary inflammation is a response to viral antigens, possibly related to the antigenicity of viral coat protein and cytotoxic T-lymphocyte responses. Time course and perivascular lymphocytic infiltrates are compatible with a response to foreign antigens. The early appearance of neutrophils is consistent with a non-specific host response. Observations have shown that bronchial epithelial cells release interleukin-8 (IL-8) after exposure to various mediators such as tumour necrosis factor (TNF), and that IL-8 acts as a neutrophil chemo-attractant thought to mediate the non-specific chronic pulmonary inflammation in CF. Replication-deficient adenoviral vectors have also been shown to increase IL-8 and ICAM-1 mRNA and protein expression in A549, a lung epithelial human cell line.[13]

*Clinical trials*

Clinical trials have been initiated in CF patients, based on nasal and/or lung directed AdCFTR in order to evaluate the safety and efficiency of gene transfer. One of these studies was performed on four CF patients in whom varying doses of CFTR recombinant adenovirus (up to $2 \times 10^9$ pfu) were administered in the nose and two days later in the lungs.[2,3] Protein and mRNA expression were documented in bronchial and nasal epithelium. Nevertheless, within 12 hours of administration of the virus, one of the subjects developed a clinical syndrome with fever, hypotension, multilobar infiltrates and a marked increase in circulating levels of the inflammatory mediator interleukin-6. No evidence of recombination or of serum neutralizing antibodies was found. This probably represented an inflammatory reaction within the lung, centred around the site of the application of the adenovirus. The symptoms resolved over a period of one month with no apparent long-term effects. No evidence of any contaminating agent was found. This subject received the highest dose of adenovirus ($2 \times 10^9$ pfu) in this study, thus it is likely that this syndrome was a dose-related phenomenon, offsetting the transfection efficiency advantage of adenoviruses.

Animal models, although showing inflammatory responses, did not predict the intensity and rapidity of the onset of this clinical syndrome, underlining the importance of such human studies. This could be explained by species differences, or by significant pre-existing lung disease observed in CF subjects. Lack of animal models of CF have limited the evaluation of gene therapy, because extrapolation to human therapy is difficult. In particular, of the animal models available, none mimics the human CF lung with significant pre-existing disease.

For some authors, human nasal studies could help predict dose related difficulties observed in lung parenchyma.[12] In fact, more extensive testing of gene transfer agents in the nasal epithelium prior to the lung do not seem helpful because it has been shown that the nasal epithelium does not mimic the responses observed in the lower airways, particularly in CF subjects. Even though the CF transmembrane conductance regulator mutations are expressed similarly in the nasal and bronchial epithelium in CF, the inflammatory consequences appear to be different, with little inflammation and no change in the proportion of epithelial cells in the nasal epithelium, compared with marked neutrophil inflammation on the epithelial surface and significant changes in epithelial populations in the large airways.[4]

Regarding the toxicity associated with administration of E1-deleted adenovirus into the airways of animals, investigations performed in a number of species, including rodents and non-human primates, showed that there is a dose dependent inflammatory response at the site of gene transfer that diminishes without permanent sequelae. Experience with intrapulmonary instillation of virus in patients is limited, but is usually unremarkable at the low doses administered to patients, except in the case of one patient. This syndrome was not predicted by the animal experiments, thus showing the importance of human studies, especially in CF. In this disorder, the lack of an animal lung model and the intense pulmonary inflammation observed complicate the evaluation of potential adenovirus vector-related toxicity.

### References

1. Brody SL, Metzger M, Danel C, Rosenfeld M, Crystal RG. Acute responses of non-human primates to the airway delivery of an adenovirus vector containing the human cystic fibrosis transmembrane conductance regulator cDNA. *Hum Gene Ther* 1994; **5**: 821–836.
2. Crystal RG, Jaffe HA, Brody SL, *et al.* A phase 1 study, in cystic fibrosis patients, of the safety, toxicity, and biological efficacy of a single administration of a replication deficient, recombinant adenovirus carrying the cDNA of the normal cystic fibrosis transmembrane conductance regulator gene in the lung. *Hum Gene Ther* 1995; **6**: 643–666.
3. Crystal RG, McElvaney NG, Rosenfeld MA, *et al.* Administration of an adenovirus containing the human CFTR cDNA to the respiratory tract of individuals with cystic fibrosis. *Nature Genetics* 1994; **8**: 42–51.
4. Danel C, Erzurum S, MacElvaney NG, Crystal RG. Quantitative assessment of human airway epithelial and inflammatory cell populations in cystic fibrosis. *Am J Crit Care Respir Dis* 1996; **153**: 362–368.
5. Mastrangeli A, Danel C, Rosenfeld MA, *et al.* Diversity of airway epithelial cell targets for *in vivo* recombinant adenovirus mediated gene transfer. *J Clin Invest* 1993; **91**: 225–234.
6. Prince GA, Porter DD, Bennett Jenson A, *et al.* Pathogenesis of adenovirus type 5 pneumonia in cotton rats (*Sigmodon hispidus*). *J Vir* 1993; **67**: 101–111.
7. Rosenfeld MA, Yoshimura K, Trapnell BC, *et al.* *In vivo* transfer of the human cystic fibrosis transmembrane conductance regulator gene to the airway epithelium. *Cell* 1992; **68**: 143–155.
8. Rosenfeld MA, Chu CS, Seth P, *et al.* Gene transfer to freshly isolated human respiratory epithelial cells *in vitro* using a replication deficient adenovirus containing the human transmembranne conductance regulator cDNA. *Hum Gene Ther* 1994; **5**: 331–342.
9. Simon RH, Engelhard JF, Yang Y, *et al.* Adenovirus-mediated transfer of the CFTR gene to lung of non-human primates: toxicity study. *Hum Gene Ther*, 1993; **4**: 771–780.
10. Welsh, MJ, Smith AE, Zabner J, *et al.* Cystic fibrosis gene therapy using an adenovirus vector; *in vivo* safety and efficacy in nasal epithelium. *Hum Gene Ther* 1994; **5**: 209–219.
11. Wilson JJ, Engelhardt JF, Grossman R, Simon RH, Yang Y. Gene therapy of cystic fibrosis lung disease using E1 deleted adenoviruses: a phase 1 trial. *Hum Gene Ther* 1994; **5**: 501–519.
12. Wilson J. Gene therapy for cystic fibrosis: challenges and futures directions. *J Clin Invest* 1995, **96**: 2547–2554.
13. Yei S, Mittereder N, Wert S, Whitsett JA., Wilmott RW, Trapnell BC. *In vivo* evaluation of the safety of adenovirus-mediated transfer of the human cystic fibrosis transmembrane conductance regulator cDNA to the lung. *Hum Gene Ther* 1994; **5**: 731–744.
